# Synthesis and Study of Organic Nanostructures Fabricated by Inclusion of 2-Methylbenzimidazole Molecules in Nanotubes of Chrysotile Asbestos, Mesoporous Silica, and Nanopores of Borate Glasses

**DOI:** 10.3390/ijms241813740

**Published:** 2023-09-06

**Authors:** Elena Balashova, Aleksandr A. Levin, Sergey Pavlov, Anatoly Starukhin, Alexander Fokin, Dmitry Kurdyukov, Daniil Eurov, Boris Krichevtsov

**Affiliations:** Ioffe Institute, Russian Academy of Sciences, Politechnicheskaya 26, 194021 Saint Petersburg, Russia; aleksandr.a.levin@mail.ioffe.ru (A.A.L.); pavlov_sergey@mail.ioffe.ru (S.P.); a.starukhin@mail.ioffe.ru (A.S.); midbarzin@yandex.ru (A.F.); kurd@gvg.ioffe.ru (D.K.); eurov@gvg.ioffe.ru (D.E.); boris@mail.ioffe.ru (B.K.)

**Keywords:** organic nanostructures, crystal structure, 2-methylbenzimidazole, FTIR, XRD, photoluminescence, dielectric properties

## Abstract

New organic nanostructures were synthesized by introducing 2-methylbenzimidazole (MBI) molecules from a melt, gas phase, or alcoholic solution into nanosized voids of borate porous glasses (PG), nanotubes of chrysotile asbestos (ChA), and mesoporous silica (MS). The incorporation of MBI into borate glasses with different pore sizes is accompanied by the appearance of several phases formed by nanocrystallites which have a MBI crystal structure, but somewhat differ in lattice parameters. The size of some crystallites significantly exceeds the size of nanopores, which indicates the presence of long-scale correlations of the crystal structure. The size of MBI nanocrystallites in ChA was close to the diameter of nanotubes (*D* ~10 nm), which shows the absence of crystal structure correlations. The XRD pattern of mesoporous silica filled by MBI does not exhibit reflections caused by MBI and a presence of MBI was confirmed only by the analysis of correlation function. The incorporation of MBI molecules into matrices is observed through optical IR absorption spectroscopy (FTIR) and photoluminescence. Introducing MBI in ChA and MS is followed by the appearance of bright green photoluminescence, the spectral structure of which is analogous to MBI crystals but slightly shifted in the blue region, probably due to a quantum-size effect. The influence of MBI inclusion in PG and ChA on the permittivity, dielectric losses, conductivity, and parameters of their hopping conductivity is analyzed.

## 1. Introduction

Modern materials science is a multifaceted field of knowledge in which fundamentally new ideas and directions are being developed, at the present stage, which are related to the creation of nanomaterials of various natures and nanosystems based on them [1]. The design of new materials, the improvement of their structure and properties, and the creation of organic and bioorganic nanomaterials and nanosystems require the use of physical diagnostic tools associated with the use of electromagnetic radiation, including from visible light to hard X-rays, methods based on the scattering of various particles such as electrons, neutrons, and ions, and as atomic resolution methods such as atomic force microscopy and atomic resolution electron microscopy, along with dielectric spectroscopy methods that allow for revealing local correlated interactions.

The main types of nanomaterials that are currently developed are thin films [2,3,4], including two-dimensional and multilayer systems obtained using the Langmuir–Blodgett technique [5], liquid suspensions [6,7], porous materials [8], and filler/matrix nanocomposites [9,10,11]. The best-known matrices used to create nanostructures of the filler/matrix type are carbon nanotubes [12,13], zeolites [14], porous borate glasses [15,16], chrysotile asbestos [17], and mesoporous silica [18,19].

In connection with the successes in the field of molecular biology and bioengineering is the prospect of creating new devices and systems for medicine, electronics, and energy, based on the possibility of embedding organic, semi-organic, or bioorganic molecules into various structures with nanosized cavities, such as nanopores, nanotubes, and voids in the crystal structure of organic and semi-organic crystals [20,21,22,23,24].

Borate mesoporous bioactive glasses have been extensively studied for biomedical applications, in particular, as drug delivery instruments and as micro-fibrous dressings to treat chronic wounds for blood vessel formation for wound healing applications and improving bone healing [25]. Mesoporous silica can also be used for drug delivery, and its particles can penetrate certain cells depending on the type of chemical coating of the particles. When the particles are filled with a fluorescent dye, they can be used as a biosensor that transports the dye through the cell membrane. Note that zeolitic imidazole ZIF-67 nanoparticles show excellent adsorption performances for organic dyes [26].

Of particular interest in this area are nanostructures based on ferroics, i.e., on ferroelectric, piezoelectric (ferroelastics), magnetic, or superconducting fillers of nanocavities [27], as well as materials with plastic phases, which make it possible to create ordered nanostructures from multifunctional materials. Scientific interest in such nanostructures may be associated with the study of size effects, in particular, changes in the physical characteristics of ferroelectrics or magnets, such as the Curie temperature, the type of phase transition, the magnitude of the magnetization or polarization of nanoparticles, and, with the emergence of new properties of multiferroics, the appearance of luminescence, as well as others. From the point of view of practical application, it seems important to study the macroscopic (bulk) magnetic, dielectric, and optical properties of such nanostructures, their dependence on the type of filler, and the possibility of their control.

This work is devoted to the synthesis and study of new filler/matrix nanocomposites fabricated by filling voids of borate porous glasses, chrysotile asbestos, and mesoporous silica with molecules of organic 2-methylbenzimidazole C_8_H_8_N_2_ (MBI). The choice of MBI as a filler was due to several reasons.

First, the relatively small size of planar heterocyclic MBI molecules (the maximum size in the plane of molecule *d*_mol_ ~0.6 nm [28]) makes it possible for them to penetrate the nanopores of matrices with a diameter *D*_pore_ > *d*_mol_. MBI crystallizes in a non-centrosymmetric lattice with monoclinic polar space group *Pn*, which is supported by ferroelectric domain switching [29] and the AFM visualization of domain wall moving under the action of an electric field [30]. Because noncentrosymmetric deformations of crystal lattices are very small and are indetectable by XRD, the MBI crystal structure is described by tetragonal group *P*4_2_/*n* (86) with the unit cell parameters *a* = 13.950(9) Å and *c* = 7.192(3) Å and a unit cell volume *V*_cell_ = 1399.6(1.4) Å^3^ [28,29]. As was expected, MBI can form nanocrystals in voids for which the volume size and diameter are higher than the *V*_cell_ and *d*_mol_, correspondingly.

Secondly, pore filling is made possible by placing the matrices in the MBI melt (*T_melt_* ~174 °C), as well as by vacuum sublimation from the gas phase, since the MBI begins to sublimate before reaching the melting temperature. In addition, as shown by this study, filling the pores of matrices is possible using a solution of MBI in alcohol at room temperature and following alcohol evaporation. Previous investigations of micron-thick MBI textured films prepared by evaporation from a solution at room temperature or by vacuum sublimation on different substrates have shown that MBI crystallizes in elongated crystallites with a needle shape, forming on substrate in-plane blocks made of splitted crystals like spherulites [31,32,33]. It is important to note that MBI has a relatively small chemical activity. The inclusion of MBI molecules cannot destroy the material of matrices or the void structure. The penetration of MBI molecules into voids can be followed only by the appearance of hydrogen bonds between ions of the matrix and MBI that cannot affect the matrix material [24]. Nevertheless, the appearance of such bonds, most probably, can influence the properties of MBI nanocrystals.

Third, bulk MBI samples demonstrate ferroelectric properties above room temperature [29,31], almost up to the melting point, because the Curie temperature of MBI at normal pressure is close to or above the melting point [29]. Spontaneous polarization *P*_s_ in MBI occurs in directions of the [110]_tetra_ type, perpendicular to the pseudotetragonal axis [001], and amounts to *P*_s_ ~5 μC/cm^2^. Dielectric hysteresis loops at room temperature show coercive fields *E*_c_ ~2–3 V/μm. An important property of MBI is the ability to switch polarization in different directions, perpendicular to the pseudotetragonal axis, by weak fields [29,31,32]. Therefore, it was interesting to see how the inclusion of MBI molecules affected the dielectric properties of the matrices.

It should also be noted that MBI, like many other organic substances, can be used in printing techniques [34]. In addition, the noncentrosymmetric crystal structure of MBI and the presence of spontaneous polarization in it makes it possible to use it to generate terahertz radiation [35].

The aim of this work was the synthesis of nanocomposites based on various matrices (borate porous glasses (PG), chrysotile asbestos (ChA), mesoporous silica (MS)) filled with organic 2-methylbenzimidazole molecules. Since the pores of borate glass, asbestos, and mesoporous silica tubes have a diameter of several nanometers, it was of interest to study the possibility of the formation of MBI nanocrystals in such systems, to study their crystal structure as well as the effect of the introduction of a filler on the infrared (FTIR) spectra, photoluminescence (PL), and dielectric properties of matrices. The description of the study samples is presented in the Section 3.

## 2. Results and Discussion

### 2.1. XRD Measurements

#### 2.1.1. MBI in Porous Borate Glasses

**Figure 1:**
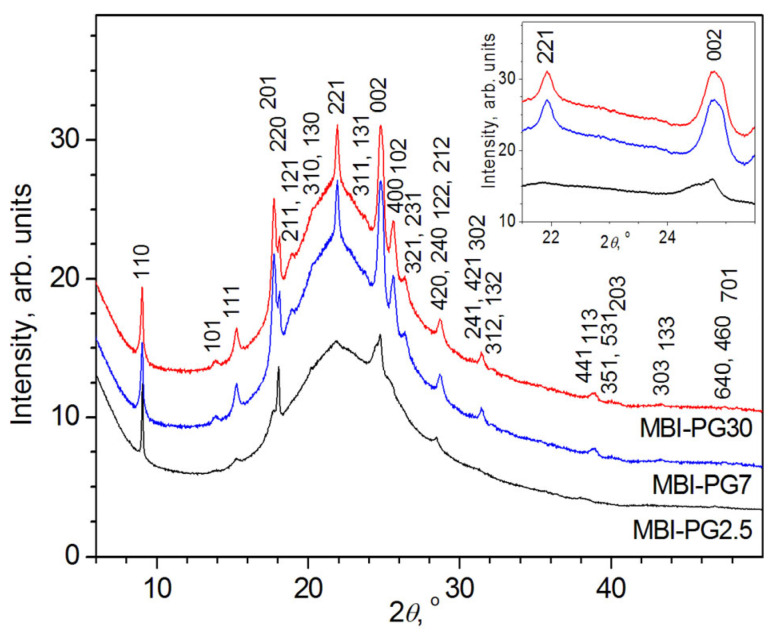
Parts of the XRD patterns of the borate glasses filled by MBI, MBI-PG2.5, MBI-PG7, and MBI-PG30. The Miller indices *hkl* of MBI XRD reflections which make the greatest contribution to the observed reflections are shown. The inset illustrates the difference in the broadening of the observed XRD with *hkl* = 221 and 002.

X-ray phase analysis showed that all reflections observed in PG with a pore diameter *D*_pore_ of ~2.5 nm, ~7 nm, or ~30 nm (PG2.5, PG7, and PG30), filled by MBI from melt (samples MBI-PG2.5, MBI-PG7, and MBI-PG30, respectively), can be attributed to the MBI crystal structure (Figure 1).

A careful inspection of the reflection profiles shows that their broadening in the samples is different. Some are very narrow, others are very wide, and in some reflections, for example, in a reflection with Miller indices *hkl* = 002, split is clearly visible. These facts indicate the presence in the samples of nanocrystallites belonging to the same crystalline phase (MBI) but having different sizes and slightly different values of the unit cell parameters and, correspondingly, unit cell volume.

- Sample MBI-PG2.5

**Figure 2:**
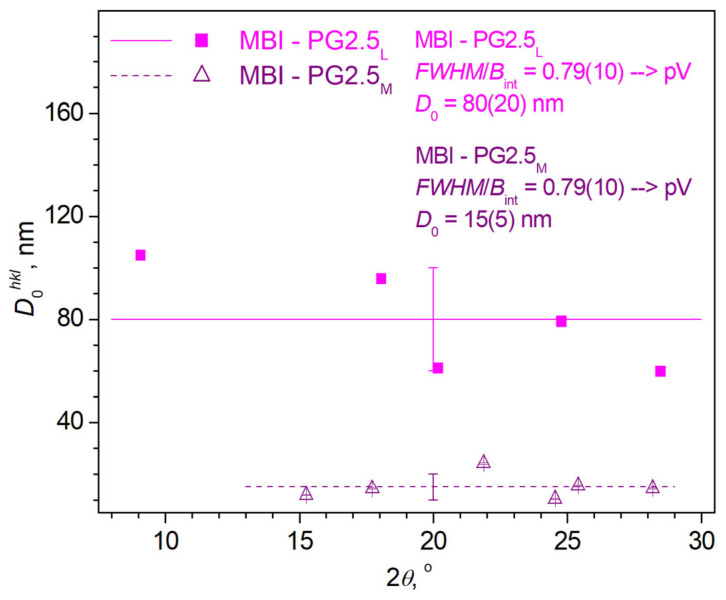
MBI-PG2.5 sample. *2θ* angle distribution of crystallite sizes, *D*_0_*^hkl^*, of MBI-PG2.5_L_ and MBI-PG2.5_M_ phases calculated in the model of zero microstrain (*ε*_s_ = 0) for observed individual reflections of the phases.

In the MBI-PG2.5 sample, the reflections are characterized by the ratio of the full-width at half maximum (FWHM) *FWHM* to the integral width *B*_int_, which lies within 0.637 ≈ 2/π < *FWHM*/*B*_int_ < (4∙ln(2)/*π*)^1/2^ ≈ 0.939 (Figure 2), i.e., they are pseudo-Voigt (pV)-type reflections [36]. Figure 2 shows the distribution of the crystallite sizes, *D*_0_*^hkl^*, of the MBI-PG2.5_L_ and MBI-PG2.5_M_ phases depending on the Bragg angles, 2*θ*_B_, of the observed individual XRD reflections (see Section 3.2.2). The horizontal lines in Figure 2 are the mean values, *D*_0_, of the phase crystallite sizes obtained by root mean square (rms) averaging of the corresponding *D*_0_*^hkl^*values.

As can be seen from Figure 2, there are two sets of reflections with narrow and wide FWHMs, which refer to crystallites with sizes of *D*_0_ ~80 nm and *D*_0_ ~15 nm. Taking into account that, according to the X-ray phase analysis, all the observed reflections belong to the MBI crystal structure, and also that the FWHM is inversely proportional to the crystallite size, these phases will be referred as MBI-PG2.5_L_ (large size) and MBI-PG2.5_M_ (mean size). The designations “mean” and “large” are used since the estimated crystallite sizes are larger than the average pore size in glass, which is ~2.5 nm.

Note that, for the MBI-PGI2.5_M_ phase, the experimental *D*_0_*^hkl^* points lie well enough on the horizontal line corresponding to the average value of *D*_0_ = 15(5) nm. In contrast to that, for the MBI-PG2.5_L_ phase, there is a systematic deviation of the experimental points from the horizontal line. The experimental values of *D*_0_*^hkl^*fall better on a straight line with a negative slope. Taking into account that the instrumental broadening has been corrected, it is obvious that such a distribution of individual values of *D*_0_*^hkl^* over the Bragg angles 2*θ*_B_ of the corresponding reflections is due to the absence of microstrains in the MBI-PG2.5_M_, in contrast to the MBI-PG2.5_L_ phase, in which the presence of microstrains is expected.

**Figure 3:**
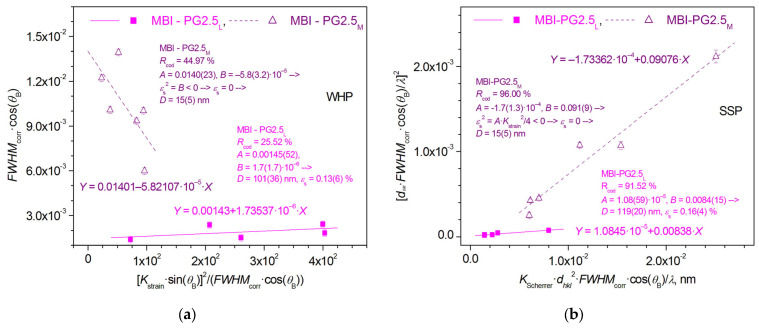
(**a**) WHP and (**b**) SSP graphs, constructed for the reflections of MBI-PG2.5_L_ and MBI-PG2.5_M_ crystalline phases of the MBI-PG2.5 sample. In (**a**,**b**), *FWHM* and *FWHM*_corr_ are FWHM values without and with correction to instrumental broadening, respectively; *K*_strain_ = 4 and *K*_Scherrer_ = 0.94 (see Section 3.2.2); *θ*_B_ is half of Bragg angle 2*θ*_B_ corrected to zero shift and displacement (Section 3.2.2); *d_hkl_* is interplane distance corresponding to XRD reflection with Miller indices *hkl* and Bragg angle 2*θ*_B_; *λ* = 0.15406 nm is wavelength of Cu-*K_α_*_1_ radiation used (after correction to Cu-*K_α_*_2_ contribution)_._ The straight approximating lines *Y* = *A* + *B*∙*X* of the WHP and SSP are shown in (**a**,**b**). Expressions for *X* and *Y* are given in axes captions of WHP and SSP graphs.

Indeed, the Williamson-Hall plot (WHP) and size-strain plot (SSP) [37] graphs, constructed using the individual reflections of MBI-PG2.5 (Figure 3), show that, instead of being grouped around a single line graph, the WHP and SSP experimental points fall into two separate regions that can be approximated by line graphs and correspond to the MBI-PG2.5_M_ and MBI-PG2.5_L_ phases.

As expected, the MBI-PG2.5_M_ phase is characterized by the absence of microstrains (*ε*_s_ = 0), whereas a non-zero microstrain level was observed in MBI-PG2.5_L_. The average crystallite size in the MBI-PG2.5_M_ phase, obtained by rms averaging of the sizes of the crystallites corresponding to individual MBI-PG2.5_M_ reflections, is *D* = *D*_0_ = 15(5) nm. The values of crystallite size and microstrain obtained from the WHP and SSP plots for the MBI-PG2.5_L_ phase are close: (*D* = 100(36)–119(20) nm, *ε*_s_ = 0.13(6)–0.16(4)%). Numerical results of the WHP and SSP calculations are shown in Table 1.

Therefore, both methods, WHP and SSP, give the same result: no contribution of microstrains to the broadening of reflections of the MBI-PG2.5_M_ phase and the presence of microstrains in the MBI-PG2.5_L_ phase.

The SSP graphs are characterized by a relatively smaller spread of experimental points around the approximating lines *Y* = *A* + *B*∙*X* compared to WHP, which is expressed by significantly higher values of the coefficient of determination *R*_cod_ = 91.52–96.00% in comparison to *R*_cod_ = 25.52–44.97% for WHP (see definition of *R*_cod_ in [37]). As a result, the *A* and *B* coefficients and, calculated from them, the *D* and *ε*_s_ values (see [37]) are characterized by smaller estimated standard deviations (e.s.d.s) in the case of SSP calculations. Therefore, the more precise values obtained during the SSP estimates will be discussed further.

An attempt at Rietveld fitting within the framework of the model of two phases of MBI with larger and smaller crystallite sizes (MBI-PG2.5_L_ and MBI-PG2.5_M_ with crystallite size ~100 nm and ~14 nm, respectively) led to a rather small value of the weighted profile factor, *R*_wp_ = 1.64% (atomic coordinates of the starting MBI model (CCDC code 1199885) were fixed for all phases during the refinement). However, the value of the weighted profile factor corrected for the background contribution, which better characterizes the quality of fitting reflection profiles, remained high, c*R*_wp_ = 27.92%. The assumption of the presence of a third phase with a small crystallite size of the order of 2.5 nm pore diameter (MBI-PG2.5_S_) led to a noticeable decrease in all the factors of agreement (*R*_wp_ = 1.36%, c*R*_wp_ = 18.86%). Despite the fact that the reflections of this MBI-PG2.5_S_ phase cannot be visualized in the XRD pattern due to the fact that they are too broad, a significant improvement in the quality of the fit indicates its presence in the MBI-PG2.5 sample.

**Figure 4:**
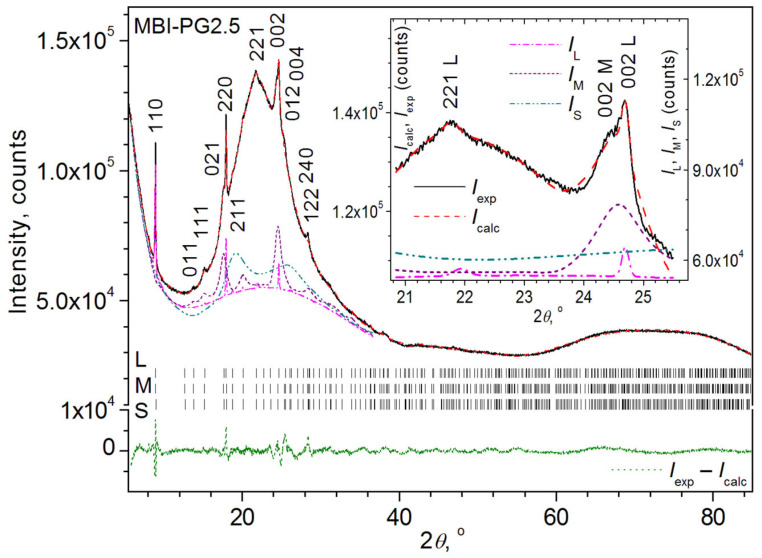
Sample MBI-PG2.5. The results of the Rietveld fit within the three-phase model MBI-PG2.5_L_ + MBI-PG2.5_M_ + MBI-PG2.5_S_. The MBI-G2.5_L_, MBI-PG2.5_M_, and MBI-PG2.5_S_ phases are designated as L, M, and S, respectively. The positions of the phase reflections calculated from the unit cell parameters refined by the Rietveld method are shown as vertical bars below. In addition to the experimental (*I*_exp_), calculated (*I*_calc_), and difference (*I*_exp_ − *I*_calc_) diagrams, the calculated XRD patterns (together with the background contribution) corresponding to individual crystalline phases are shown in the region 2*θ* = 6–37°. The Miller indices *hkl* of some selected observed reflections are indicated. The inset shows, on an enlarged scale, part of the diagrams in the region of reflections 221 and 002 of the MBI phases.

The results of the Rietveld fitting for the three-phase model (MBI-PG2.5_L_ + MBI-PG2.5_M_ + MBI-PG2.5_S_) are graphically illustrated in Figure 4 and summarized in Table 1.

As can be seen from Table 1, all MBI phases are characterized by close unit cell parameters. However, the unit cell volume, *V*_cell_, of the MBI-PG2.5_M_ phase is close to the *V*_cell_ of the MBI compound described in the CCDC databank (CCDC code 1199885). At the same time, the MBI-PG2.5_L_ and MBI-PG2.5_S_ phases are characterized by ~1–2% larger unit cell volumes. The sizes of the crystallites of the MBI-PG2.5_L_ and MBI-PG2.5_M_ phases (respectively, *D*^TOPAS^ = 107(1) nm and 12(1) nm, as well as microstrain *ε*_s_^TOPAS^ = 0.082(1)% for the MBI-PG2.5_L_ phase) obtained in the Rietveld refinement agree well with the values obtained in the SSP and WHP calculations, but are characterized by a smaller e.s.d.s. The refinement results in the size of the crystallites of the MBI-PG2.5_S_ phase, *D*^TOPAS^ = 2.4(2) nm, being almost equal to the pore size of the sample MBI-PG 2.5. The weight content of the MBI-PG2.5_S_ phase with the smallest crystallite size is the largest, being 73.7(7) wt.%. At the same time, the weight content of the MBI-PG2.5_L_ phase with the largest crystallite size is the smallest, only 0.80(2) wt.%. The remaining 25.5(7) wt.% of the weight content of the sample corresponds to the MBI-PG2.5_M_ phase with an intermediate crystallite size.

Thus, various methods lead to the fact that the sizes of crystallites are not uniformly distributed around a mean value (Figure 2 and Table 1). The crystallite sizes of two phases (MBI-PG2.5_L_ and MBI-PG2.5_M_) significantly exceed the diameter of the *D*_pore_ pores, ~2.5 nm, and the size of the crystallites of the MBI-PG2.5_S_ phase is approximately the value of *D*_pore_. The presence of crystallites with a size that is considerably higher than the size of the pores may be explained by taking into account two factors. The first one is that, for the *θ*–2*θ* scan mode used in our measurements, the XRD measures the size of the crystallite in the direction that is perpendicular to the diffracting interatomic planes, i.e., perpendicular to the surface of the glass sample. The second factor is that the pores in PG2.5 are curved hollow cylinder-like fibers that are distributed arbitrarily, relative to the surface of the sample.

MBI in the parts of the pores lying parallel to the surface of the glass sample will form MBI-PG2.5_S_ crystallites, and MBI in the parts of the pores perpendicular to the sample surface or inclined to it will lead to the formation of MBI-PG2.5_L_ and MBI-PG2.5_M_ crystallites with larger sizes. Obviously, if the porous glass was not prepared in a special way, there cannot be many areas which result in the formation of large crystallites. As a result, the larger the crystallite size, the lower weight content of the corresponding MBI phase, and, accordingly, the smaller the relative volume occupied by the phase.

Since the MBI-PG2.5 XRD patterns (Figure 1 and Figure 4) contain reflections characterized by different Miller indices *hkl* (110, 011, 111, etc.), the crystallites of different MBI phases in the pores do not have exactly the same orientation. Nevertheless, as the Rietveld fit (Table 1) and comparison with the theoretical XRD pattern of MBI (cf. Figure 4 and Figure S1 of Section S1.1 of Supplementary Materials, simulated using structure data of CCDC 1199885 card by means of program PowderCell v.2.4 [39]) show, most of the crystallites of the MBI-PG2.5_L_, MBI-PG2.5_M_, and MBI-PG2.5_S_ phases are oriented along the crystallographic directions [110], [001], and [211], respectively (i.e., the crystallographic planes (110), (001), and (211) in the crystallites of the corresponding phases lie preferentially parallel to the sample surface).

It should be noted that the sizes of the crystallites of the phases MBI-PG2.5_L_ and MBI-PG2.5_M_ are much larger than the pore diameter in glass, which indicates that the crystal structure of MBI is correlated over a large number of pores. Correlation manifests itself not only in the parameters of the MBI unit cell, but also in the orientation of the MBI crystal axes, discussed above, since each phase has its preferential orientation, described by the March-Dollase parameter, *r*_MD_ (see Table 1). The appearance of large areas with a correlated crystal structure and orientation of the filler (i.e., crystallites) has also been observed in different nanosystems [40,41,42].

- Sample MBI-PG7

The analysis of the XRD reflection profiles using the WHP and SSP methods shows that, for the MBI-PG7 sample, it is possible to distinguish three characteristic average sizes of crystallites which correspond to three MBI phases, MBI-PG7_L_, MBI-PG7_M1_, and MBI-PG7_M2_ (see Section S1.2 and Figures S2 and S3a,b of Supplementary Materials and Table 2).

The crystallite sizes of these phases are ~60 nm (MPB-PG7_L_ phase), ~28 nm (MPB-PG7_M1_ phase), and ~17 nm (MPB-PG7_M2_ phase). However, the Rietveld fitting indicates the presence of a fourth phase, MPB-PG7_S_, with an even smaller average crystallite size. The assumption of the presence of the MBI-PG7_S_ phase with crystallite sizes equal to or smaller than the pore sizes in the MBI-PG7 sample led to a significant decrease in the agreement factors, to *R*_wp_ = 1.64% and *cR*_wp_ = 13.02% from *R*_wp_ = 2.53% and c*R*_wp_ = 30.40%.

**Figure 5:**
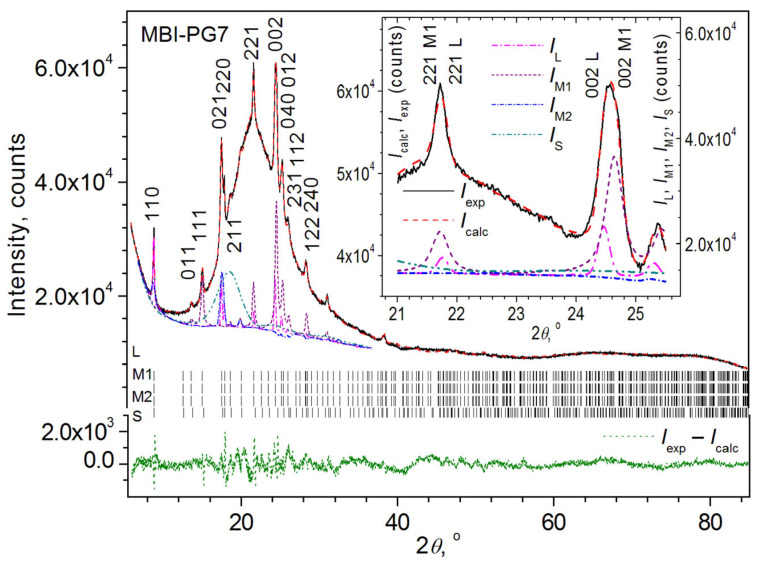
Sample MBI-PG7. Results of the Rietveld fit within the four-phase MBI-PG7_L_ + MBI-PG7_M1_ + MBI-PG7_M2_ + MBI-PG7_S_ model. The MBI-PG7_L_, MBI-PG7_M1_, MBI-PG7_M2_, and MBI-PG7_S_ phases are designated as L, M1, M2, and S, respectively. Other details are the same as in caption for Figure 4.

The graphical results of the Rietveld fit carried out within the framework of the four-phase model (MBI-PG7_L_ + MBI-PG7_M1_ + MBI-PG7_M2_ + MBI-PG7_S_) are shown in Figure 5. The quantitative results are summarized in Table 2.

The average sizes of the crystallites of the phases MBI-PG7_L_, MBI-PG7_M1_, and MBI-PG7_M2_ (*D*^TOPAS^ = 61(3) nm, 28(1) nm, and 17(1) nm, respectively) obtained in the Rietveld refinement coincide well with the values of *D* obtained by the SSP and WHP methods (Table 2), and are characterized by smaller e.s.d.s. The value of the microstrain of the crystallites of the MBI-PG7_L_ phase (*ε*_s_^TOPAS^ = 0.014(6)%) obtained in the refinement also agrees satisfactorily with the SSP and WHP data (Table 2), taking into account its e.s.d. Refinement by the Rietveld method led to a small size of the crystallites of the MBI-PG2.5S phase, *D*^TOPAS^ = 2.7(1) nm. As in the case of the MBI-PG2.5S phase with approximately the same small size of crystallites in the MBI-PG 2.5 sample, the broadening of the reflections of this phase is large, and they are visually indistinguishable in the XRD pattern. Nevertheless, the significant improvement in the quality of the fit described above evidences the presence of this phase. According to our quantitative estimates, when refined by the Rietveld method, the weight contents of phases in the composition of the MBI-PG7 sample are greater the smaller their size is (71.33(14) wt.%, 9.71(17) wt.%, 5.70(9) wt.%, and 0.80(2) wt.% for phases MBI-PG7_L_, MBI-PG7_M1_, MBI-PG7_M2_, and MBI-PG7_S_, respectively). Thus, the mass content of the MBI-PG7_L_ and MSI-PG7_S_ phases with the largest and smallest crystallite sizes, respectively, and the total mass content of the MBI-PG7_M1_ and MBI-PG7_M2_ phases with intermediate crystallite sizes, are approximately the same as for the similar MBI-PG2.5_L_, MSI-PG2.5_S_, and MBI-PG2.5_M_ phases in the MBI-PG2.5 sample. Like the phases of the MBI-PG2.5 sample, all of the crystalline phases of the MBI-PG7 sample exhibit close unit cell parameters. In comparison to CCDC code 1199885 MBI, the MBI-PG7S phase is characterized by a ~1% smaller unit cell volume, *V*_cell_, whereas the *V*_cell_ of all of the other phases is ~1–1.5% larger.

Table 1 and Table 2 show that the relative volume, *V_i_*, occupied by a certain phase, *i*, in the MBI-PG2.5 and MBI- PG7 samples is roughly inversely proportional to the crystallite size *D*_i_ (*V_i_*~*D_i_*^−1^). Since the volume occupied by phase *i* is *V_i_* = *N_i·_v_i_*, where *N_i_* is the number of crystallites of phase *i*, and *v_i_*~*D_i_*^3^ is the volume of a crystallite of phase *i*, we can assume that the number of crystallites of phase *i* is *N_i_*~*D_i_*^−4^.

Thus, the WHP and SSP analysis of the XRD reflection profiles and Rietveld fitting of the XRD pattern leads to a model of the MBI-PG7 sample that is similar to the model of the MBI-PG2.5 sample of the MBI phases with close unit cell parameters and large, intermediate, and small crystallite phases. The only difference is that there is not one crystalline phase with an intermediate crystallite size, as in MBI-PG2.5, but two phases (MBI-PG7_M1_ and MBI-PG7_M2_). In addition, for the MBI-PG7_S_ phase, the smallest crystallites are characterized by sizes smaller than the pore size *D*_pore_ ~7 nm, in contrast to MBI-PG2.5, where the smallest crystallites are approximately equal to the pore size. As discussed above, the weight contents of the MBI-PG7 phases follow the same tendency, as in MBI-PG2.5, i.e., for the larger crystallite size, a lower weight content of the corresponding MBI phase is observed. Therefore, for PG2.5, we can assume the same model of formation of the MBI phases in the pores of the PG7, which are curved, hollow, cylinder-like fibers that are arbitrarily distributed relative to the sample surface, resulting in the appearance of large areas with a correlated crystal structure and orientation of the MBI filler. As in the MBI-PG2.5 sample, the microstrain is observed in the MBI-PG7_L_ phase, apparently due to the large size of the crystallites of this phase.

In contrast to MBI-PG2.5, in the MBI-PG7 sample, most of the crystallites of the MBI-PG7_L_, MBI-PG7_M1_, MBI-PG7_M2_, and MBI-PG7_S_ phases are oriented along the crystallographic directions [001], [012], [241], and [211], respectively. The preferential orientation of the phases with the smallest crystallite size is the same as in MBI-PG7 and MBI-PG2.5 samples.

It is interesting to note the complete similarity of the XRD reflection profiles in the samples MBI-PG7 and MBI-PG30 (Figure 1), which is probably due to the formation of MBI crystalline phases in these samples with similar cell parameters, sizes, and orientations of the crystallites.

#### 2.1.2. MBI in Chrysotile Asbestos

Two types of chrysotile asbestos (ChA) samples filled by MBI from melt were investigated. The first sample, designated as the MBI-ChA sample, was a plate of ~10 × 10 × 1 mm^3^ filled with MBI, where ChA fibers in the form of hollow tubes with a diameter of ~9 nm passed in one direction along one of the long sides of the plate and parallel to the surface of the plate. Another one, referred to as MBI-ChA-mill, was the sample MBI-ChA milled into powder.

X-ray phase analysis shows that all observed XRD reflections of the MBI-ChA and MBI-ChA-mill samples are attributed to the MBI structure. Line profile analysis of the XRD reflections using WHP and SSP techniques (Figures S4 and S5a,b in Section S1.3 of Supplementary Materials) results in a MBI crystallite size of about the diameter of the ChA tube, *D*^SSP^ ≈ *D*^WHP^ = 8.0(2.5) nm, and microstrain *ε*_s_^SSP^ = *ε*_s_^WHP^ = 0.

Analysis of the XRD patterns of the MBI-ChA and MBI-ChA-mill samples was performed using the Le Bail (LB) fitting method [43], which allows for fitting without using a structural model and, correspondingly, neglecting the influence of the preferential orientation of crystallites.

However, the assumption of only one MBI-ChA_1_ phase did not allow us to obtain a sufficient LB fit of the XRD pattern of the MBI-ChA sample. For example, the reflection profile with Miller indices *hkl* = 130 (Bragg angle 2*θ*_B_ ≈ 21.6°) did not fit properly, and the weighted profile agreement factor was only *R*_wp_ = 12.46%.

The LB fitting showed that the best correspondence to the experiment (*R*_wp_ = 3.58%) was achieved when two phases, MBI-ChA_1_ and MBI-ChA_2_, were present in the MBI-ChA sample, which have slightly different values of unit cell parameters and crystallite sizes *a* (~14.06 and 13.63 Å) and *c* (7.16 and 7.13 Å). The same was obtained for the powder sample MBI-ChA-mill (*R*_wp_ = 3.87%).

**Figure 6:**
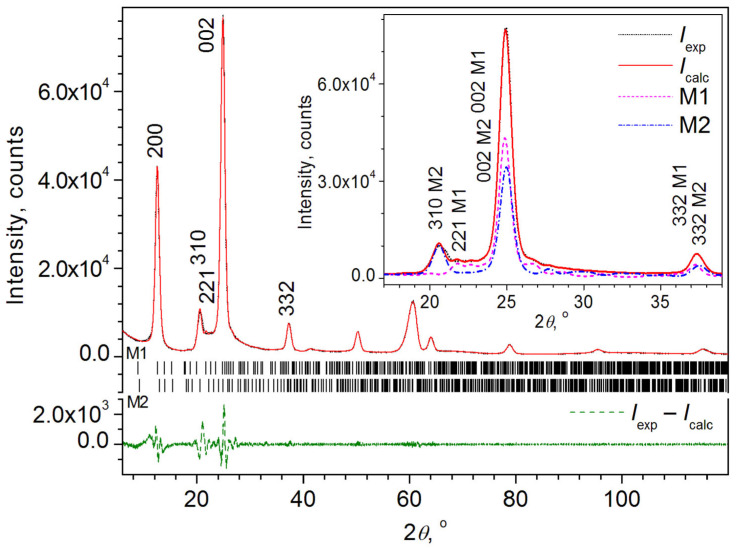
Sample MBI-ChA. Results of the LB fit in frames of the two-phase MBI-ChA1 + MBI-ChA_2_ model. The MBI-ChA_1_ and MBI-ChA_2_ phases are designated as M1 and M2, respectively. Other details are the same as in the caption to Figure 4. The positions of the phase reflections calculated from the unit cell parameters refined by the LB method are shown as vertical bars below. The experimental, calculated, and difference diagrams are marked as *I*_exp_, *I*_calc_, and *I*_exp_ − *I*_calc_, respectively. The Miller indices, *hkl*, of some selected observed reflections are indicated. The inset shows the experimental and calculated XRD patterns and calculated diagrams (together with the background contribution) corresponding to individual crystalline phases in the region 2*θ* = 17–39°.

The final graphical results of the LB fitting for MBI-ChA are shown in Figure 6, and for MBI-ChA-mill are presented in Figure S6 of Supplementary Materials.

Table 3 shows the characteristics of these phases that were obtained from LB fitting for both the bulk MBI-ChA and milled MBI-ChA-mill samples.

It should be noted that milling did not lead to the appearance of spherical particles, and the elongated MBI particles were still stacked mainly along the tubular pores of ChA, so the results of both measurements turned out to be very close (cf. results for MBI-ChA and MBI-ChA-mill in Table 3). The crystallite sizes of the MBI-ChA_1_ and MBI-ChA_2_ phases in both samples are close to ~9 nm, which coincides with the pore diameter of chrysotile asbestos. The MBI-ChA_1_ phase occupies about two times more volume of the MBI-ChA and MBI-ChA-mill samples than the MBI-ChA_2_ phase.

The values of the unit cell parameters in both phases of the samples are close to those observed in bulk crystal (CCDC code 1199885). However, the unit cell parameter *a* in both phases is smaller than the tabular value, whereas the value of parameter *c* in the MBI-ChA_1_ phase is slightly larger and, in the MBI-ChA_2_ phase, it is noticeably smaller than in the bulk crystal. This is also reflected in the unit cell volume *V*_cell_ (Table 3), resulting in a ~1% larger value of *V*_cell_ in the MBI-ChA_1_ phase and, vice versa, a ~5% smaller value in MBI-ChA_2_.

Thus, the incorporation of MBI into ChA is also accompanied by the appearance of MBI phases. However, unlike porous glasses, MBI-ChA contains only two phases. The size of the MBI crystallites of these phases is close to the diameter of ChA nanotubes (*D*_pore_ ~9 nm), evidently because the ChA nanotubes run parallel to each other and the surface of the ChA sample, and the XRD (*θ*–2*θ* scan mode) measures the size of the crystallite in the direction that is perpendicular to the surface of the ChA sample. Phases with larger or smaller crystallites were not found in MBI-ChA. This means that there are no correlations of the MBI crystal structure between different chrysotile asbestos nanotubes.

The close but nevertheless different crystallite sizes of the MBI-ChA_1_ and MBI-ChA_2_ crystalline phases could be explained by two possible models. In the first case, the diameter of the ChA nanotubes is the same throughout the sample and is approximately equal to the maximum size of the crystallite (phase MBI-ChA_1_, *D* ≈ 9.7 nm averaged by MBI-ChA and MBI-ChA-mill samples). The phase MBI-ChA_2_ is characterized by a smaller crystallite size, *D* ≈ 8.75 nm (value, averaged by MBI-ChA and MBI-ChA-mill samples), since it does not fill the entire volume of the hollow ChA nanotube. In the second case, probably closer to reality, there are two characteristic close diameters of the nanotube in the ChA sample which is completely filled with MBI, *D*_pore_ ≈ 9.7 nm and *D*_pore_ ≈ 8.75 nm.

Visual inspection of the measured and the simulated theoretical MBI XRD patterns (Figure S6 of Supplementary Materials and Figure 6 in comparison with Figure S1 of Supplementary Materials) leads to the conclusion that the samples are strongly influenced by the effects of preferential orientation. The preferential orientation effects in the ChA samples filled with MBI are much stronger than those in porous glasses (for example, reflection 110, 021, etc. are not seen in the XRD patterns of the ChA samples (cf. Figure S6 of Supplementary Materials and Figure 6 comparing with Figure 4 and Figure 5)), which is expected from the location of the ChA nanotubes in the ChA samples. Consideration of the results of the LB fitting indicates that the main preferential directions of the MBI-ChA_1_ and MBI-ChA_2_ phases are different. For the MBI-ChA_1_ phase, the directions are [100] and [001], and for the MBI-ChA_2_ phase, they are [001] and [013].

#### 2.1.3. MBI in Mesoporous Silica (MBI-MS)

In contrast to the MBI-PG2.5, MBI-PG7, and MBI-ChA bulk-plate samples, the sample of mesoporous silica (MS), with a cylindrical pore diameter of *D*_pore_ ~3 nm and filled with MBI (referred as MBI-MS), was a powder. In contrast to the MBI-PG2.5, MBI-PG7, MBI-ChA, and MBI-ChA-mill samples, the XRD patterns of the MBI-MS sample do not show the presence of reflections attributed to MBI or any other phase. Only a halo due to the amorphous SiO_2_ matrix is observed (Figure S7 in Section S1.4 of Supplementary Materials).

Therefore, to detect MBI, total correlation functions *c*(*r*) were built for the experimental XRD patterns of the MBI-MS and pure MS according to the method from [44] (Section 3.2.5 and Section S2 of Supplementary Materials).

**Figure 7:**
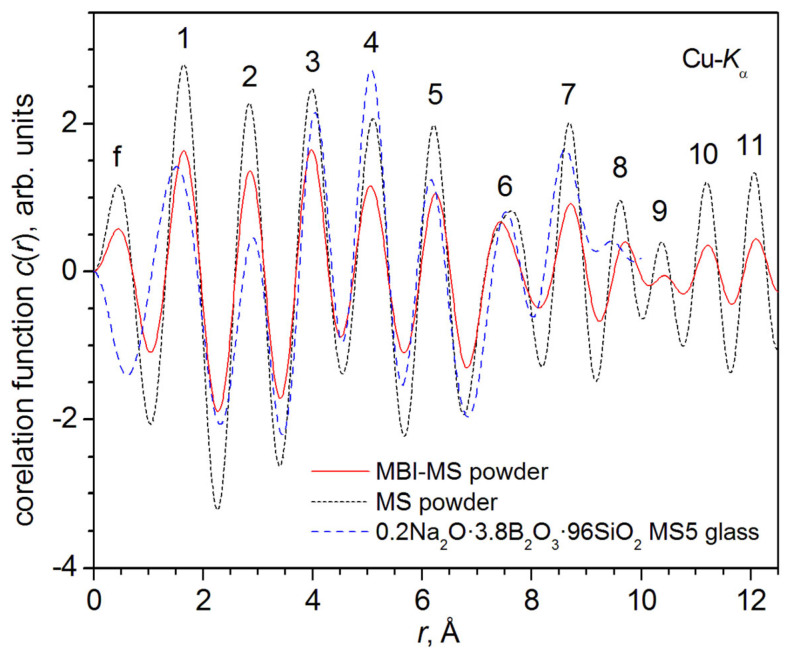
Total correlation functions *c*(*r*) built from the measured XRD patterns of MBI-MS and pure MS. For comparison, the *c*(*r*) function for mesoporous silica MS5 glass with composition 0.2Na_2_O·3.8B_2_O_3_·96SiO_2_ is shown. Designations of the figure are explained in text. Numbers indicate the correlation maxima and correspond to mean distances in silica structure. Symbol f indicates the false peak arising due to the breakage of the Fourier series during calculations of *c*(*r*).

In Figure 7, the symbol f indicates a false peak, which arises due to the breakage of the Fourier series. The numbers indicate the correlation maxima, the positions of which correspond to the average distances between atoms in the amorphous material under study and can be correlated with the average interatomic distances in the crystalline material of the same composition.

The positions of the *c*(*r*) maxima, i.e., the observed correlation distances for MBI-MS and pure MS, coincide well with each other and with the positions of the sodium-borate mesoporous alumina oxide (MS5) glass with a pore size of *D*_pore_ ~5 nm and a composition of 0.2Na_2_O·3.8B_2_O_3_·96SiO_2_ (mol.%) from [44].

The peaks of the correlation function *c*(*r*) of MBI-MS are characterized by lower heights (intensities) and being somewhat wider than the *c*(*r*) of pure MS. In [44], the wider *c*(*r*) peaks of lower intensity were also observed for natrium-borate bulk glass 18.1Na_2_O∙16.9B_2_0_3_∙65SiO_2_ (mol.%) in comparison with MS5 glass. It was concluded that this is caused by the greater disorder of the local bulk glass structure compared to MS5. It is also possible that MBI-MS is characterized by a greater disorder of the local structure compared to pure MS due to the entry of MBI into the pores of the MBI-MS sample.

Thus, in contrast to MBI-ChA and MBI-PG, there are no reflections on the MBI-MS XRD pattern. Apparently, this is due to the relatively low filling of the MS nanotubes with MBI filler and the lack of correlation of the MBI structure in neighboring MS nanotubes. As a result, the MBI-MS nanocrystallites are very small and, consequently, the XRD reflections are very broad. Nevertheless, studies have shown that the correlation functions, *c*(*r*), in MBI-MS are characterized by lower intensities and are somewhat wider than in pure MS, which may indicate the presence of MBI nanocrystallites.

### 2.2. FTIR and Photoluminescence Measurements

#### 2.2.1. IR Absorption in ChA and MBI-ChA Nanostructure

**Figure 8:**
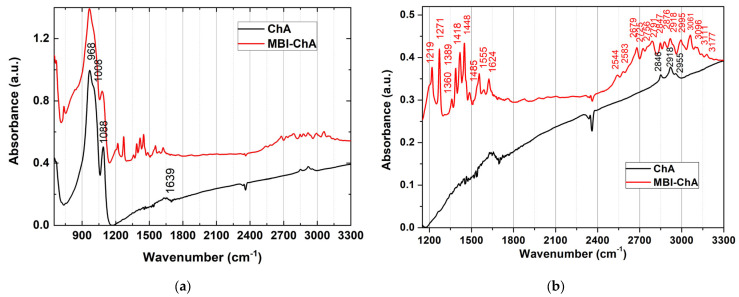
(**a**) IR absorption spectra of samples of “pure” ChA and MBI-ChA. (**b**) Enlarged image of the IR absorption spectra of the samples in the wavenumber region *ν* = 1150–3300 cm^−1^. Spectra are shifted vertically for clarity.

To obtain IR absorption spectra, FTIR measurements were performed on thin fibers split from a bulk MBI-ChA sample. The measurements were carried out in the transmission mode. The methods of reflection and frustrated total reflection did not give the best results. The spectrum of MBI-ChA, shown in Figure 8 (see also Table 4), is consistent with the literature data on IR absorption in MBI crystals [45,46] and in ChA [47].

The main absorption peaks of ChA are observed in the spectral region *ν* = 900–1200 cm^−1^. Three modes at *ν* = 986, 1008, and 1088 cm^−1^, usually attributed to the Si–O valence modes, were observed in our samples. The peak at 1088 cm^−1^ corresponds to the stretching vibrations of Si-O bonds in the Si-O-Si system [47]. The wider intense bands at 986 and 1008 cm^−1^ are associated with antisymmetric vibrations in the Si-O-Si complexes.

In the MBI-ChA sample, in addition to ChA peaks, two groups of lines in the ranges of 1200–1750 cm^−1^ and 2500–3200 cm^−1^ are observed in the spectrum, and are associated with vibrations in the MBI molecules [45,46]. Table 4 shows the positions of the main absorption peaks and their interpretations from [46].

A broad band at *ν* = 1635 cm^−1^ that was observed earlier in ChA in Reference [47] is also presented in our sample at 1639 cm^−1^ (Figure 8). Note that this band has also been observed in some crystals containing water and was attributed to bending vibrations of the water molecules [48,49,50]. The bands at 2846, 2918, and 2993 cm^−1^ also refer to stretching vibrations of water [48]. The position of water stretching vibrations indicates strong hydrogen bonds and the participation of water molecules in various hydrogen bond structures [48]. The appearance of water vibrations in the absorption spectrum of ChA indicates the presence of water molecules in the pores of the ChA.

Thus, the IR absorption spectrum clearly shows the presence of MBI molecules in the MBI-ChA sample. Since the MBI lines in the IR spectrum are associated mainly with the internal vibrations in the MBI molecule, which are only slightly affected by the environment, the resonance energy and line width are practically the same as in the MBI crystal or in the MBI solution. In the MBI-PG and MBI-MS samples, IR absorption from the MBI was not observed in the FTIR experiments because of very high contribution to absorption from the matrixes.

#### 2.2.2. Photoluminescence of MBI Single Crystals

**Figure 9:**
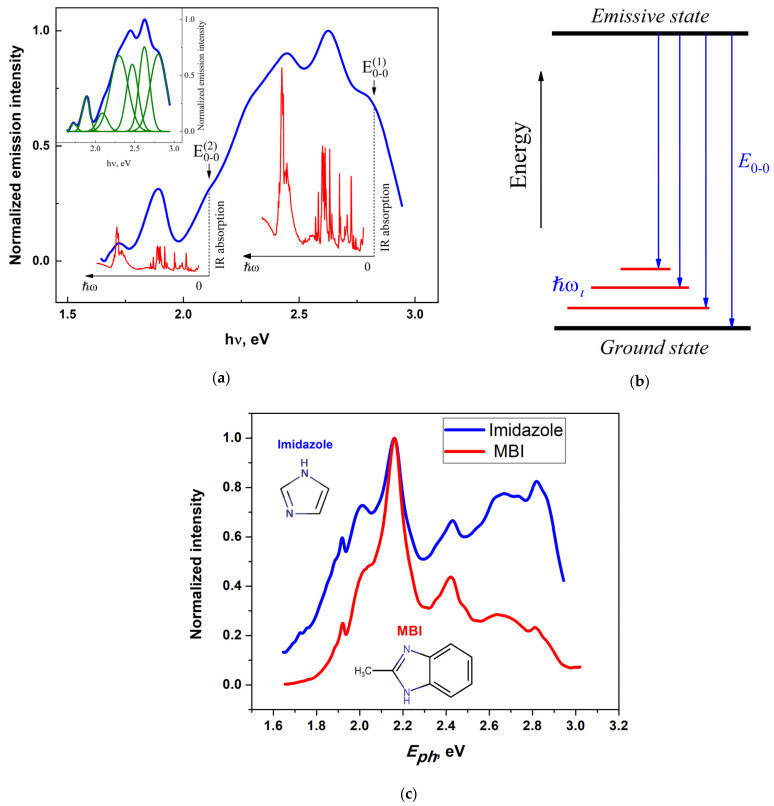
(**a**) The emission spectrum of a bulk MBI crystal (blue line) upon excitation by light with a wavelength of 405 nm (*E*_photon_ = 3.061 eV). Measurement temperature *T* = 293 K. The insets show the decomposition of the MBI emission contour into Gaussian bands (olive lines) and fragments of the IR absorption spectra of MBI (red lines). (**b**) A scheme of vibronic transitions in an emission center. (**c**) Luminescence spectra of imidazole and MBI crystals upon excitation by light with a wavelength of 405 nm (*E*_photon_ = 3.061 eV). Measurement temperature *T* = 80 K.

The bulk MBI crystals under study were prepared by the condensation of MBI vapor. The crystals were almost colorless, with a slight beige tinge, and with an average volume of about 1 mm^3^. The room temperature luminescence spectrum of the crystals upon excitation with 405 nm light (*E*_photon_ = 3.061 eV) is shown in Figure 9.

The spectrum consists of a series of broad overlapping bands occupying almost the entire visible region. To our knowledge, the energy gap, *E*_g_, in MBI crystals is unknown. On the other hand, a sharp increase in optical absorption in the MBI solution in ethanol, which can be related to the highest occupied molecular orbital-lowest unoccupied molecular orbital (HOMO-LUMO) transitions in MBI molecules, was found to start at a photon energy with *hν* ≥ 3.8 eV. Apparently, this value can be considered in the first approximation as an estimate of the *E*_g_ value in MBI crystals. Therefore, the emission of MBI crystals observed in the region *hν* < 3 eV (Figure 9a) should be ascribed to the recombination of free or localized excitons (hereafter referred to as emission centers). The observed structure of the emission spectrum can be due to electron transitions from excited emissive states in the MBI crystal to vibrationally excited substates of the ground electronic state (Figure 9b).

In such a case, an emission spectrum is expected to consist of the band of a purely electronic transition at energy *E*_0−0_ and its vibrational replicas at energies *E_i_* = *E*_0−0_ − *ħω_i_*, where *ħω_i_* is the energy of the *i*-th vibration (phonon) involved in the transition (Figure 9b). The spectral contour of MBI emission shown in Figure 9a indicates the presence of at least seven overlapping bands (inset in Figure 9a). Figure 9a also shows the IR vibrational spectra of MBI (according to [45]). As can be seen, there is an obvious correlation of the positions of the vibronic emission bands with the vibrational spectrum of the crystal. The best agreement between the considered model of vibronic optical transitions and the vibrational spectrum of the crystal is achieved by assuming the existence of two emission centers (emissive states) in the crystal with energies of purely electronic transitions, $E_{0-0}^{\left(1\right)}$ and $E_{0-0}^{\left(2\right)}$ (Figure 9a).

The MBI molecule consists of imidazole and benzene rings (see inset in Figure 9c). In studying this connection, it is of undoubted interest to understand the role of these rings in the formation of the emission spectrum of the molecule. The emission spectra of imidazole (image of imidazole molecule is shown in Figure 9c) and MBI crystals at low temperatures turned out to be the most indicative in this respect. As can be seen from Figure 9c, the structures of the low-temperature spectra of these crystals are almost identical. This suggests that the emission spectrum of MBI crystals is most likely formed mainly by electronic transitions in the imidazole ring. The detailed interpretation of the MBI low-temperature emission spectrum will be given elsewhere.

#### 2.2.3. Photoluminescence of MBI-ChA

**Figure 10:**
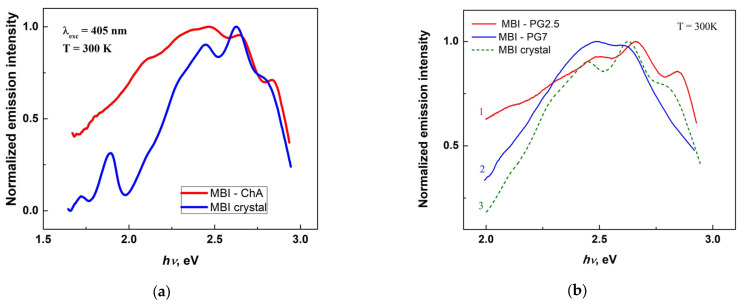

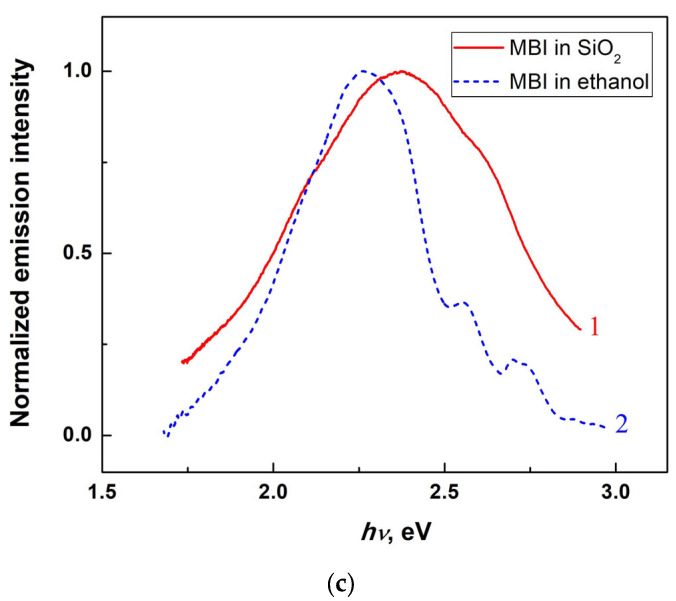
(**a**) Emission spectra of MBI nanoparticles in MBI-CA (red line) and MBI bulk crystal (blue line) upon excitation by light with a wavelength of 405 nm (*E*_photon_ = 3.061 eV). Measurement temperature *T* = 300 K. (**b**) Emission spectra of MBI nanoparticles in porous borate glasses with pores of 2.5 nm (1) and 7 nm (2) and MBI bulk crystal (3) upon excitation by light with a wavelength of 405 nm (*E*_photon_ = 3.061 eV). Measurement temperature *T* = 300 K. (**c**) PL spectra of MBI in mesoporous silica (1) and in ethanol solution (2) under 405 nm light excitation. Measurement temperature *T* = 300 K.

“Pure” ChA is practically nonluminous at room temperature. The introduction of MBI into the pores of asbestos is accompanied by the appearance of quite bright photoluminescence, the spectrum of which is represented in Figure 10. It is known that, compared with bulk crystals, nanocrystals have a larger fraction of surface atoms and, accordingly, an increased role of surface states, which often serve as channels for the radiationless deactivation of electronic excitations and thereby contribute to a decrease in the photoluminescence intensity. The relatively bright luminescence of MBI nanoparticles in ChA shows that the effect of such states on the luminescence intensity of MBI nanocrystals is small.

As in the case of pure MBI crystals, the short-wavelength part of the MBI emission spectrum in MBI-ChA is well structured and similar to the structure of the spectrum of pure MBI. It is also noticeable that the bands in the spectrum of MBI in asbestos are broader than in the emission spectrum of the crystals and are shifted to the shorter wavelength side by ~16 meV.

A slight shift of the emission spectrum of MBI nanoparticles in asbestos to a shorter wavelengths relative to the spectrum of bulk MBI crystals can be attributed to the quantum-size effect in MBI nanoparticles. This effect has been theoretically investigated in nanoclusters of some polycyclic aromatic hydrocarbons [51]. It was shown that, in such nanoclusters, the HOMO-LUMO gap increases with a decreasing number of molecules in the nanocluster. In this case, the observed broadening of the bands in the spectrum of nanoparticles can be explained by the inhomogeneous broadening of the spectrum due to the MBI-nanoparticle size dispersion in ChA.

#### 2.2.4. Photoluminescence in Porous Borate Glasses Filled with MBI

As in the case of asbestos, the introduction of MBI into porous borate glasses is accompanied by the appearance of bright photoluminescence. The room-temperature photoluminescence spectra of glasses with 2.5 and 7 nm pores filled with MBI are shown in Figure 10b. Comparison of the spectra depicted in Figure 10b shows that the spectrum of the MBI-PG2.5 glass composite is very close to that of the bulk crystal. The small “blue” shift in the composite spectrum with respect to the crystal spectrum can be attributed to the nanoparticle-size effect mentioned above. The position of the emission bands in the 7 nm glass, where the larger pore size allows for the formation of larger nanoparticles, already coincides with the position of the spectral bands in the bulk crystal. The large pore size in 7 nm glass also appears to contribute to the relatively large dispersion of MBI nanoparticle sizes, resulting in significant inhomogeneous broadening of its emission spectrum (Figure 10b).

#### 2.2.5. Photoluminescence of Mesoporous Silica Filled with MBI

The photoluminescence spectrum of MBI introduced into mesoporous silica with ~3 nm pores is shown in Figure 10c. The spectrum consists of a broad, weakly structured band with a maximum at *hν*_max_ ≈ 2.370 eV (*λ*_max_ ≈ 507 nm). Note that the luminescence intensity of MBI in MS is weaker than that of MBI in asbestos and borate glasses. The absence of a pronounced structure makes it difficult to directly compare the emission spectrum of MBI nanoparticles in silica with the spectrum of bulk MBI crystal. The difference in the shape of the spectrum of MBI in porous silica from the above-described emission spectra of MBI in asbestos and porous glasses, as well as MBI crystals, can indicate the presence of a specific interaction between the MBI molecules and silica matrix. Due to the synthesis conditions of mesoporous silica, its surface contains a significant number of hydroxyl groups that are capable of forming hydrogen bonds with MBI molecules on the nanoparticle surfaces, which affects the electronic states of the MBI molecules and the vibrational spectrum of the associates.

The perturbations of the electronic and vibrational spectra are expected to be different for MBI molecules in different microenvironments in the pores of an MS matrix. This leads to an additional inhomogeneous broadening of the emission spectrum of the nanoparticle ensemble, which is also characteristic of solutions. In this connection, it can be noted that the shape of the spectrum of MBI in mesoporous silica appears to be close to the shape of the emission spectrum of MBI solution in ethanol (Figure 10c), which is also a proton donor. At the same time, the perturbation of the spectra is relatively small, so that the main emission still falls on the green region of the spectrum, as in the case of the emissions of MBI in ChA and in borate glasses.

The formation of hydrogen bonds between MBI molecules and the MS matrix can also account for the relatively weak luminescence of MBI in MS, which is caused by the effect of hydrogen bonds on the rate of the radiationless deactivation of the molecules. Another reason for the lower intensity of the emission of MBI in the MS matrix is the lower content of MBI per unit volume of the MS matrix compared to its content in ChA and borate glasses.

### 2.3. Dielectric Properties

#### 2.3.1. MBI-ChA

**Figure 11:**
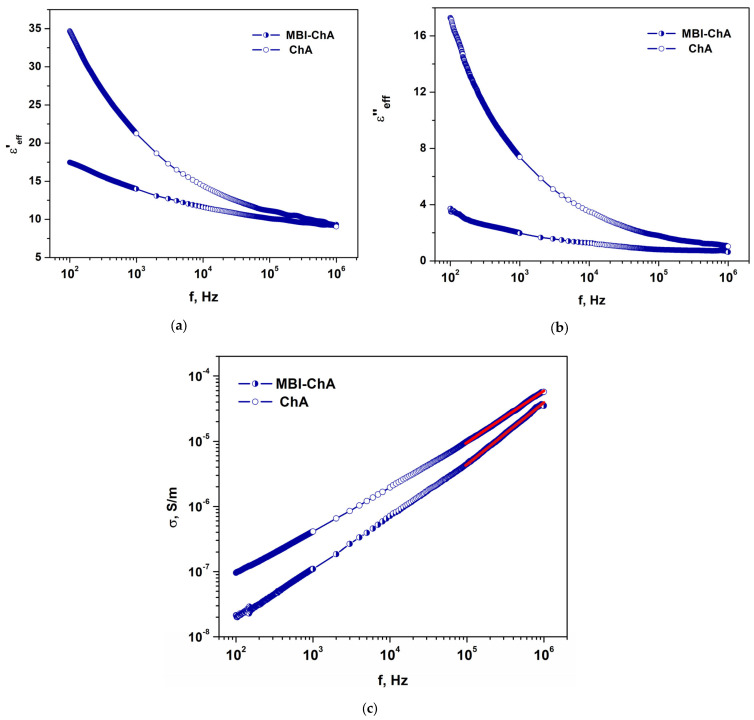
Frequency dependencies of effective permittivity. (**a**) *ε*′_eff_, (**b**) *ε*″_eff_, and (**c**) conductivity *σ* in MBI-ChA and ChA.

Figure 11 shows the frequency dependencies of the real and imaginary parts of the effective permittivity, *ε*′_eff_ and *ε*″_eff_, of the ChA and MBI-ChA samples, measured in the frequency range *f* = 25–10^6^ Hz for an electric field applied perpendicularly to the nanotubes. In the low-frequency region (*f* ~10^2^–10^4^ Hz), we see considerable differences in the values of both the real and imaginary parts of the effective permittivity, *ε*′_eff_ and *ε*″_eff_, of the samples. The introduction of MBI in nanotubes of ChA leads to a significant decrease in the effective dielectric constant, *ε*′_eff_, and *tgδ*. In particular, at *f* = 100 Hz, the *ε*′_eff_ and *tgδ = ε*″_eff_/*ε*′_eff_ in MBI-ChA are almost two times smaller than in the “empty” ChA.

Large values of *ε*′*_eff_* and *tgδ* in ChA samples at low frequencies are associated with the response of molecules, in particular water molecules, adsorbed in nanotubes from the air to a driving electric field [52]. With the introduction of MBI, the permittivity of ChA-MBI approaches the values observed in MBI crystals [31], which indicates the absence of water molecules in the nanotubes.

At high frequencies (*f* ~10^5^–10^6^ Hz), the values of *ε*′_eff_ both in ChA and MBI-ChA are almost the same, *ε*′_eff_ ≈ 10. This is due to the fact that the permittivities in MBI crystals (*ε*′ ~8) and ChA *(ε*′ ~10) are very close in this frequency range [53]. At *f* = 1 MHz, the values of the *ε*″_eff_ and dielectric losses in MBI-ChA and ChA are also close. The value of *tgδ* in MBI-ChA (*tgδ* ≅ 0.08) is even lower than in ChA (*tgδ* ≅ 0.12) (Figure 11b). This is because, at a high frequency, the contribution of water molecules is considerably reduced.

Figure 11c shows the frequency dependencies of conductivity (*σ = ωε*′*ε*_0_∙*tgδ*) in the MBI-ChA and ChA samples in a double logarithmic scale. At low frequencies, the conductivity of MBI-ChA is close to that of MBI crystals and much lower than that of ChA. The large values of ChA conductivity are due to the adsorption of water molecules in the nanotubes. In both samples, strong frequency dependencies on conductivity are observed, including in the low frequency region. This indicates that the direct current (DC) conductivity is low and does not prevail over the alternating current (AC) conductivity even at low frequencies (*f* ~100 Hz). At high frequencies, the slopes of frequency dependence on conductivity in ChA and MBI-ChA have different values: 0.8 and 0.94, respectively.

#### 2.3.2. MBI in PG

**Figure 12:**
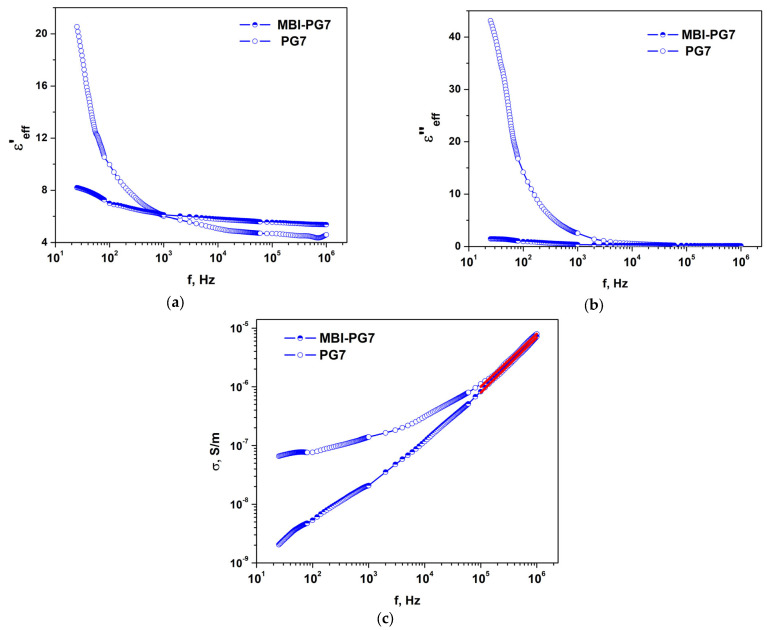
Frequency dependencies of the real and imaginary parts of effective permittivity. (**a**) *ε*′_eff_, (**b**) *ε*″_eff_, and (**c**) conductivity *σ* in PG7, MBI-PG7 samples.

Figure 12 shows the frequency dependencies of the effective permittivity, *ε*′_eff_—the real part of the dielectric constant (a), imaginary part of dielectric constant, *ε*″_eff_, (b), and conductivity, *σ*, (c) in the porous glass samples PG7 and MBI-PG7. Similar to the case of the ChA and MBI-ChA samples, significant differences between the porous glass samples without MBI (PG7) and the samples with MBI included in the pores (MBI-PG7) are observed in the low-frequency region. The effective permittivity, *ε*′_eff_ and *ε*″_eff_, are much higher in the porous glass without MBI. The permittivity, *ε*′_eff_ and *ε*″_eff_, is significantly reduced when MBI is introduced into glass nanopores. The values of the real *ε*′_eff_ and imaginary part *ε*″_eff_ of the effective permittivity in MBI-PG7 are close and even somewhat lower than in MBI crystals, which indicates the absence of water molecules in the pores.

At high frequencies, the value of the effective permittivity of PG7 is lower than in the MBI-PG7 samples, which confirms the inclusion of MBI in the pores of the glass, since the value of the permittivity of glass without pores is *ε*′ ≈ 5, which is almost half the permittivity of MBI crystals.

At low frequencies (*f* = 10–10^4^ Hz), the magnitude and frequency dependencies of the conductivity in the PG7 and MBI-PG7 samples show significant differences (Figure 12c). For *f* < 100 Hz, the conductivity in PG7 is practically frequency independent, which indicates the prevalence of DC conductivity with a value of *σ_DC_* ≈ 8⋅10^−8^ S/m. In MBI-PG7, the conductivity at low frequencies is much lower and strongly depends on the frequency. This indicates that the regime of dominance of DC conductivity is not achieved. At a high frequency, the conductivity in both of the samples shows close frequency dependencies, with a slope of 0.90 ± 0.01 in PG7 and 0.94 ± 0.01 in MBI-PG7.

**Figure 13:**
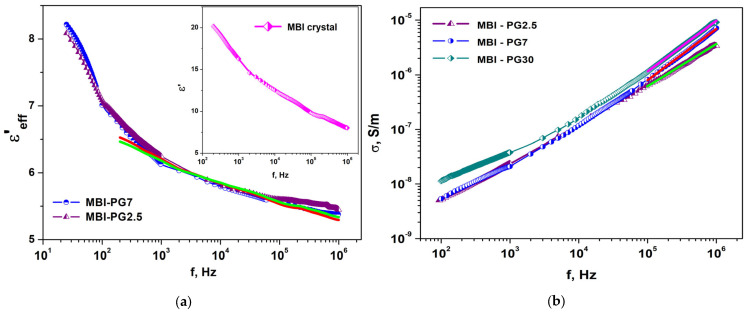
Frequency dependencies of the (**a**) real part of effective permittivity, *ε*′_eff_, in MBI-PG7 (blue) and MBI-PG2.5 (purple), and (**b**) conductivity *σ* in MBI-PG7 (blue), MBI-PG2.5 (purple), and MBI-PG30 (green) samples. Inset in (**a**) shows frequency dependence of permittivity *ε*′_eff_ in MBI crystals along [110] direction. Red and green lines in (**a**) show results of calculations.

To show the effect of pore size on the dielectric characteristics of porous glasses filled with MBI, measurements were carried out on samples with pore sizes of 2.5 nm (MBI-PG2.5), 7 nm (MBI-PG7), and about 30 nm (MBI-PG30). Frequency dependencies of the real part of the effective permittivity, *ε*′_eff_, in MBI-PG7, MBI-PG2.5 (a), and conductivity, *σ*, (b) in the MBI-PG7, MBI-PG2.5, and MBI-PG30 samples are shown in Figure 13. The inset shows the frequency dependence of permittivity, *ε*′_eff_, in the MBI crystals along the [110] direction.

**Figure 14:**
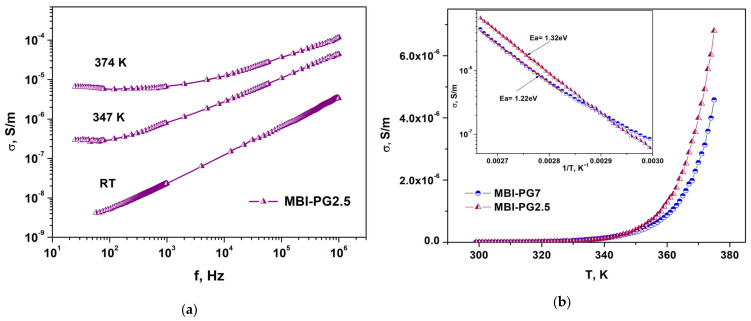
Frequency dependencies of the conductivity, *σ*, in MBI-PG2.5 at different temperatures (**a**) and temperature dependencies of conductivity, *σ*, in MBI-PG7 and MBI-PG2.5 samples for *f* = 60 Hz (**b**). Inset in (**b**) shows dependence of conductivity, *σ*, on inverse temperature in MBI-PG7 and MBI-PG2.5 samples.

Figure 14 presents frequency dependences of the conductivity, *σ*, in MBI-PG2.5 at different temperatures (a) and temperature dependencies of conductivity, *σ*, in the MBI-PG7 and MBI-PG2.5 samples for *f* = 60 Hz (b). The inset shows the dependence of the conductivity, *σ*, on the inverse temperature in the MBI-PG7 and MBI-PG2.5 samples.

#### 2.3.3. Calculations

For calculations of effective permittivity in the MBI-PG7 sample, we used the theoretical expressions of the dielectric response of ferroelectric-dielectric composites (Equation (1) [54] and Bruggerman equation (Equation (2) [55]) for dielectric composites obtained in the effective medium approximation (EMA). The first approach considers the composite formed by the dielectric matrix with permittivity *ε*_d_, volume concentration *q* and ferroelectric inclusions with permittivity *ε*_f_, and gives the following expression for effective permittivity, *ε*_eff_:

*ε*_eff_(*q*) = ¼[−*ε*_d_ + 3*qε*_d_ + 2*ε*_f_ − 3*qε*_f_ + (8*ε*_d_*ε*_f_
*+* (−*ε*_d_ + 3*qε*_d_ + 2*ε*_f_ − 3*qε*_f_)^2^)^1/2^]. (1)

The second approach gives the possibility of calculating the *ε*_eff_ of a composite formed by *N* components with a volume concentration *q_j_*, and with a permittivity *ε*_d*j*_, by solving the equation:

(2) $$\sum_{j=1}^{N}q_{j}\cdot \frac{\varepsilon_{eff}-\varepsilon_{dj}}{\varepsilon_{dj}+2\cdot \varepsilon_{eff}}=0,$$

In calculation, we used the values of the dielectric constant and the volume of PG matrix, *ε*_d_ = 5 and *q* = 0.75–0.8, respectively.

The calculated effective permittivity, *ε*_eff_ for Equations (1) and (2) is shown in Figure 13a as red and green lines, correspondently. The calculations were carried out using the experimental dependencies of *ε*′_eff_ for the MBI crystal shown in the inset of Figure 13a and the value of the permittivity for glass without pores *ε*_d_ = 5. It can be seen from Figure 13a that a good agreement between the experimental and calculated values of *ε*_eff_ in the MBI-PG7 and MBI-PG2.5 samples was achieved. In Equation (2), we also take into account, besides glass and MBI, empty pores with a volume of about 3% on the sample volume. This gives a somewhat better description of the experimental data. It should be noted that, during preparation, the entire pore volume is occupied by the MBI melt, but small empty volumes may appear due to a decrease in the volume of MBI after crystallization.

Equations (1) and (2) show that the effective permittivity is determined by the total volume of pores in the sample, and not by their linear size. Samples MBI-PG7 and MBI-PG2.5 are characterized by approximately the same values of permittivity, as follows from Figure 13a. This indicates that, at the same permittivities of the matrix and filler, the effective permittivity of the composite is determined only by the volume occupied by the components, which is practically the same in MBI-PG7 and MBI-PG2.5. The same pore volume for both MBI-PG7 and MBI-PG2.5 is determined only by the same composition of borate glass used to create PG7 and PG2.5 nanoglasses. The calculated dependencies of the effective permittivity obtained within the framework of the models used are consistent with the experimental data.

The AC conductivity of a material, *σ_AC_* = *ωε*′*ε_0_tgδ* = *ωε*″*ε_0_* (where *ε*_0_ is the permittivity of a vacuum), is usually described by the expression [56,57]:

*σ_AC_* = *σ_D__C_* + *Aω ^s^*, (3)

where *σ_D__C_* is the DC conductivity, *ω* = 2π*f* is the circular frequency, *A* is the constant, and *s* is the exponent of frequency dependence. In the case of hopping conductivity 0.5 < *s* ≤ 1 [56,57].

At room temperature, only in the PG7 sample, the conductivity has no frequency dependence at low frequencies (Figure 12c) and *σ_D__C_* ≈ 8⋅10^−8^ S/m. For MBI-ChA and MBI-PG7, the conductivity has a strong frequency dependence in the studied frequency range, which means that the DC conductivity mode is not achieved *(σ_DC_* << *σ_AC_*) (Figure 11c and Figure 12c).

At high frequencies, *f* ~ 10^5^–10^6^ Hz, in all samples, *σ* is frequency dependent. Linear approximation of *σ(f)* dependence in the range *f* = 0.1–1 MHz gives the value of the exponent as *s* = 0.80 in ChA, *s* = 0.94 in MBI-ChA, and *s* = 0.90 and 0.94 for PG7 and MBI-PG7, correspondingly (Figure 11c and Figure 12c). The non-unity in the exponent values may indicate specific conductivity mechanisms [58,59]. The most probable of these seems to be hopping charge transfer between localized states separated by an energy barrier. The correlated barrier hopping (CBH) model considers carrier hopping between barrier-separated states that are randomly distributed in the sample volume [58]. This model was successfully used to describe the low frequency conductivity in thin films of scandium oxide [58], as well as in betaine phosphate crystals with a 5% addition of BPI [59]. The value of the *s* parameter makes it possible to estimate the energy difference *W_m_ =* 6*kT*/*(*1 *− s)* between the ground state and the free state in which a carrier can travel over the lattice. The described calculations yield *W_m_* ≅ 2.58 eV for the MBI-ChA nanostructure and *W_m_* ≅ 0.77 eV for the ChA. The values of *W_m_* and *ε* can now be used to determine the Bohr radius, *a*, of a localized carrier, *a* = *e*^2^/(2*εW_m_*) ≈ 4.4 Å for the MBI-ChA and 12 Å for ChA. Therefore, the incorporation of MBI molecules in a ChA matrix results in a considerable change in parameters of the charge carrier hopping mechanism. For PG7 and MBI-PG7 nanostructures, our calculations give closer values: *W_m_* ≅ 1.55 eV and 2.58 eV, and *a* ≈ 5.1 Å and 7.3 Å, respectively. Since the glass used for the preparation of PG has a low dielectric constant and very small dielectric losses, the frequency dependencies of the conductivity and dielectric losses of MBI-PG7 are caused mainly by the MBI component.

To reveal the influence of the pore size on the frequency dependencies of the conductivity, samples of porous glass with the inclusion of MBI with pore sizes of 30 nm, 7 nm, and 2.5 nm were studied. The frequency dependencies of conductivity for MBI-PG2.5, MBI-PG7, and MBI-PG30 samples are shown in Figure 13c. The value of the exponent *s* in MBI-PG7 is *s* = 0.94 ± 0.01 and, in MBI-PG30, *s* = 0.91 ± 0.01. These values are close and are considerably higher than *s* = 0.79 ± 0.01, which was observed for MBI-PG2.5. In MBI-PG2.5, *W_m_* ≅ 0.74 eV and *a ≈* 18.1 Å. Note that, in this case, the carrier localization radius approximately corresponds to the radius of the pores filled with MBI.

The frequency dependencies of the conductivity in the MBI-PG7 and MBI-PG2.5 samples at different temperatures are shown in Figure 14. In MBI-PG2.5 (and MBI-PG7), for measurement temperature *T* > 247 K, the conductivity at low frequencies is frequency independent. This means that the conductivity at low frequencies is determined by σ_DC_, which increases with the temperature (Figure 14a). The linear fitting of conductivity frequency dependences at high frequency reveals a decrease in exponent s with a temperature increase that is in agreement with the CBH model [58].

In both the MBI-PG7 and MBI-PG2.5 samples, an increase in temperature above 340 K results in a drastic increase in the DC conductivity, *σ*_DC_, which make it possible to calculate the activation energies, *E_a_* (Figure 14b). The inset in Figure 14b shows the dependencies of the conductivity, *σ*, on the inverse temperature in these samples. The calculated values of *E_a_* in the MBI-PG7 and MBI-PG2.5 samples are equal to 1.22 eV and 1.32 eV, respectively. These activation energies for DC conduction are higher than the *E_a_* ≈ 1.1 eV found in an MBI film [31], which is characteristic of proton conduction in crystals with chains of hydrogen bonds [60]. The parameters *s*, *W_m_*, *a*, and *E_a_* for the ChA, MBI-ChA, PG, and MBI-PG structures are shown in Table 5.

In all samples, the exponent *s* is less than one. In MBI-ChA, PG7, MBI-PG7, and MBI-PG30, in which the sizes of the pores are *D*_pore_ ≥ 7 nm, the values of *s* are close and vary from *s* = 0.90 to *s* = 0.94. In MBI-PG2.5, the *s* is considerably smaller: *s* = 0.79. This is also reflected in the smaller values of *W_m_* and the larger carrier localization, *a*.

## 3. Materials and Methods

### 3.1. Sample Preparation

**Figure 15:**
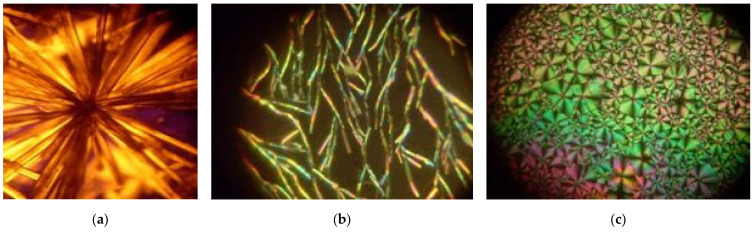

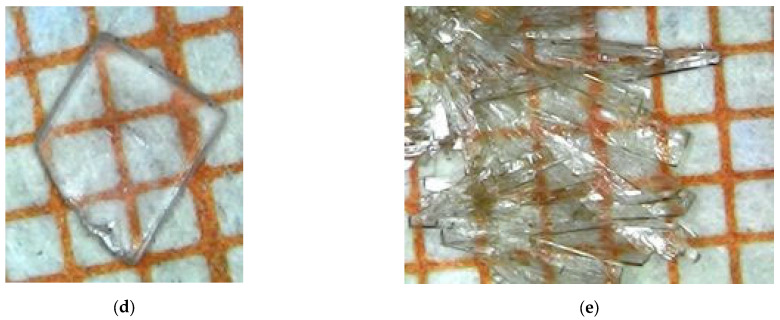
Images of MBI crystals prepared by different methods. (**a**) Split crystals of the spherulite type, consisting of elongated crystallites growing radially in an ethanol solution along pseudotetragonal axes [001]_tetr_ from one crystallization center. (**b**) Dendrite MBI crystals prepared by sublimation of gas phase on glass surface. (**c**) Image of textured MBI film grown by evaporation of MBI ethanol solution on NdGaO_3_ substrate in crossed polarizers. (**d**) MBI crystals grown by vacuum sublimation from gas phase. (**e**) MBI crystal from acetone solution.

Organic nanostructures used in this work were synthesized by introducing MBI molecules into porous borate glasses, chrysotile asbestos, and mesoporous silica. MBI crystals used for nanostructure preparation were grown by evaporation, as well as by slow cooling from saturated solutions in ethanol or acetone (or deuterated acetone (d-acetone)) [61] of MBI powder obtained by chemical methods. The solution was purified with activated carbon. To obtain more perfect crystals, they were repeatedly recrystallized. Also, MBI crystals were obtained from the gas phase by vacuum sublimation. Examples of MBI crystals grown by different methods are shown in Figure 15 along with image of an MBI film with a thickness of ~5 µm obtained by evaporation on substrate.

Depending on the growth conditions, MBI crystals can have different shapes, such as split crystals of the spherulite type, formed in an ethanol solution by elongated crystallites growing radially along the [001]_tetra_ pseudotetragonal axes from one crystallization center (Figure 15a); dendritic-type crystals after sublimation of MBI from the gas phase onto a Pt/glass substrate (Figure 15b); MBI films several microns thick obtained by evaporating an ethanol solution of MBI (Figure 15c), consisting of self-organizing spherulitic blocks [31,32]. Bulk MBI crystals can be prepared from acetone (or an alcohol) solution (Figure 15e) or from the gas phase (Figure 15d).

Description of preparation methods and properties of various types of porous glasses can be found in [62,63,64]. A typical example of porous silica glass is Vycor glass (Corning 7930) with standard chemical composition: 96% SiO_2_, 3% B_2_O_3_, 0.40/a Na_2_O, R_2_O_3_ ± RO_2_ < 1% (R = Al_2_O_3_ or ZrO_2_). It is made by acid leaching the boron-rich phase in a phase-separated borosilicate glass. The result is a high-content (96%) silica glass containing an interconnected network of pores typically less than 100 Å in diameter. In our experiments, we used Vycor glasses with average pore diameter of *D*_pore_ ~2.5 nm, ~7 nm, and ~30 nm which was evaluated by nitrogen adsorption and/or desorption measurements, and a standard porosimetry analysis.

For introducing MBI into pores of Vycor glasses, the templates of glasses with dimensions of ~5 × 5 × 0.5 mm^3^ were placed in MBI melt (*T*_melt_ ~174 °C) for several days without any external pressure. After removing from the melt and cooling up to room temperature, the samples were mechanically cleaned from tailings of MBI remaining on the surface of the matrix. The analysis of sample weight before and after MBI introduction shows ~80% filling of pores by MBI.

**Figure 16:**
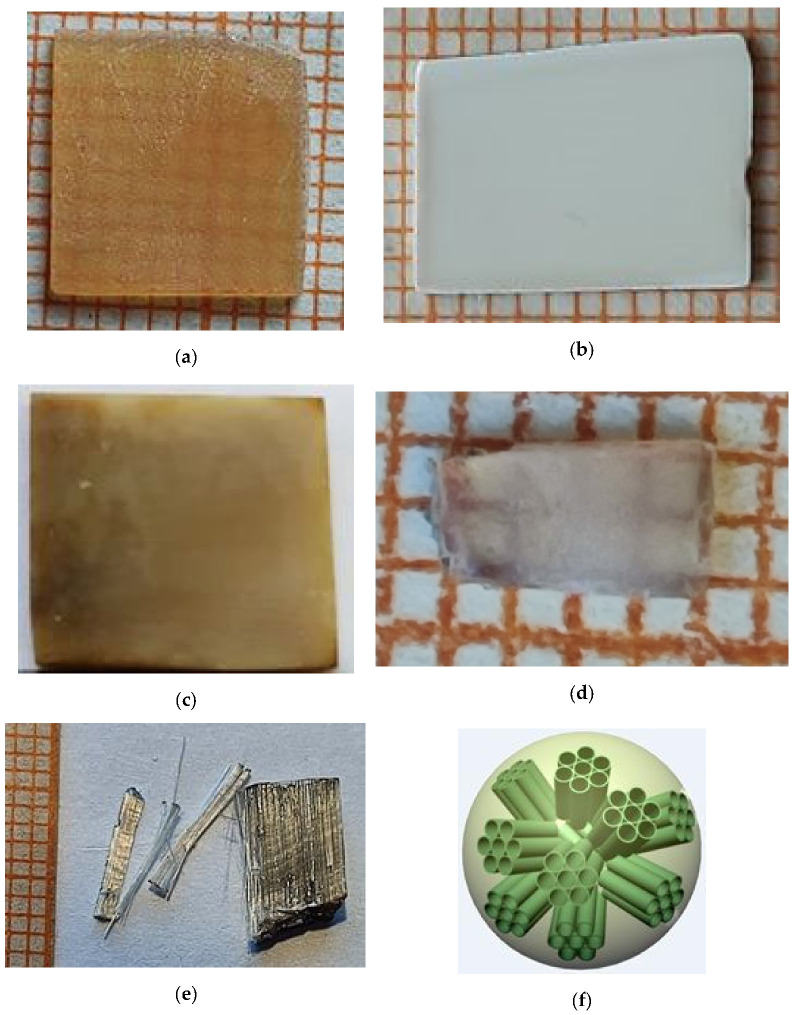
Photos of porous glasses and asbestos filled by MBI. (**a**) Porous glass with *D*_pore_ ~7 nm pores size without MBI. Porous glass with diameter of pores (**b**) *D*_pore_ ~30 nm, (**c**) ~7 nm, and (**d**) ~2.5 nm filled by MBI from melt. (**e**) Images of chrysotile asbestos matrix (tube diameter of ChA, *D*_pore_ ~9 nm) filled by MBI from melt. Split asbestos fibers were used for IR absorption (FTIR) measurements. Size of samples ~(8–12) × (6–10) × 1 mm^3^. (**f**) Schematic representation of the location of nanopores in a silica microparticle.

Also, the introduction of MBI into borate glasses was carried out from the gas phase. For this purpose, MBI crystals and a Vycor glass template were placed in an evacuated and sealed quartz tube and heated to a temperature below *T*_melt_. A few days later, the matrix was filled with MBI at 80% filling of pores. Photos of porous glass before and after filling with MBI are shown in Figure 16a–d.

Chrysotile asbestos, Mg_3_Si_2_O_5_(OH)_4_—magnesium hydrosilicate, refers to layered silicates. In the case of chrysotile, the layers are twisted into tubules with an inner diameter of 30–60 Å and an outer one, on average, 300–400 Å. Chrysotile asbestos fibers form a hexagonal, dense packing. The walls of the tubes are formed by about 20 double-ribbon layers. One layer consists of silicon-oxygen tetrahedra with hydroxyl groups in the vertex plane. Three oxygen atoms of these tetrahedra are shared with neighboring tetrahedra. The second layer is located on the tops of these tetrahedra. It is composed of hydroxyl groups and magnesium ions and twists the double-ribbon layer into a hollow tube. There are up to 20 such double layers in the wall. Chrysotile asbestos with the inclusion of 2-methylbenzimidazole was prepared by placing and impregnating an asbestos template in an MBI melt. We used natural chrysotile asbestos with a tube diameter of *D*_pore_ ~9 nm (Figure 16e).

An important advantage of monodisperse spherical mesoporous silica particles (MSMPs) is the presence of an internal system of cylindrical nanochannels of the same diameter (controllably varied within 2–5 nm, on average ~2.5 nm) with a volume of up to 60% of the particle volume (Figure 16f). MSMPs were synthesized via basic hydrolysis of tetraethoxysilane in a water-ethanol-ammonia mixture containing cylindrical micelles of a surfactant pore-forming agent, cetyltrimethylammonium bromide. The synthesis procedure is described in detail in [65]. The molar ratio of the reagents tetraethoxysilane: NH_3_:H_2_O:C_2_H_5_OH was 1:60:370:230. The synthesis duration was 1 h. The particles obtained were centrifuged, dried in air at 80 °C for 24 h, and calcined at 550 °C for 5 h. The average diameter of the particles was 510 ± 30 nm, pore size was 3.1 ± 0.2 nm, pore volume was 0.48 cm^3^/g (~50% of particle volume), specific surface area (BET) was 800 m^2^/g.

To introduce MBI into the pores of silica particles, a capillary impregnation method was used. For this, a weighed portion of SiO_2_ particles was impregnated with alcohol solution of MBI under ambient conditions, and then dried at 60 °C. The amount of the solution and concentration of MBI were chosen so that, after embedding into the pores of silica particles, its weight content was 10%. After impregnation of MBI content (determined gravimetrically) into obtained MBI-MS (MS filled with MBI), powder sample was found to be 9.8 wt.%, which, taking the density of the filler to be close to 1 cm^3^/g, amounted to ~20% of the pore volume.

### 3.2. Details of X-ray Diffraction Experiment and Analysis

#### 3.2.1. Measurement of XRD Patterns

XRD measurements were carried out on a D2 Phaser X-ray powder diffractometer (Bruker AXS, Karlsruhe, Germany) using an X-ray tube with a copper anode, monochromatized with a Ni filter (Cu-*K_α_* radiation). A LYNXEYE (Bruker AXS, Karlsruhe, Germany) semiconductor linear X-ray detector was used to record XRD patterns.

The measurements were carried out in the symmetrical scanning mode *θ*-2*θ* (2*θ* is diffraction angle and ω = 2*θ*/2 = *θ* is the angle of incidence of X-rays on the surface of the sample) in the vertical Bragg-Brentano *θ*-*θ* geometry. To reduce the influence of the possible effect of the preferential orientation of crystallites, during measurements, the sample was rotated around the axis of the holder, which coincided with the axis of the diffractometer goniometer.

The XRD patterns of the MBI-PG2.5, MBI-PG7, and MBI-PG30 samples for phase analysis and Rietveld fitting were recorded in the range of 2*θ* = 6°–85° with a step of Δ2*θ* = 0.02°. The XRD patterns of the MBI-ChA and MBI-ChA-mill containing more intensive reflections were recorded in a wider range of 2*θ* = 6°–120°. The amorphous-like XRD pattern of the MBI-MS was measured in maximum possible range 2*θ* = 5.6°–142° to reduce possible Fourier series breakage errors when constructing a correlation function.

To correct the diffraction patterns for counter zero shift (Δ2*θ*_zero_) and to correct (during calculations) the angular positions of the reflections for a shift due to a possible misalignment with the focal plane (Δ2*θ*_displ_), additional measurements were carried out. For these measurements, the plate samples were embedded in the Si640f X-ray powder standard (NIST, Gaithersburg, MD, USA) in such a way that the surface of the plates was at the same level with the surface of the Si powder, and both the sample surface and the surface of the Si powder fell into the X-ray beam during measurements. The Si reflections recorded on these XRD patterns were used as an internal standard to correct the angular positions of the reflections from the corresponding samples measured together with the standard. In turn, the obtained corrected angular positions of reflections with a strong intensity from the samples were used as an external standard for correcting the angular positions of the reflections of the samples in subsequent measurements without use of the Si640f standard.

X-ray phase analysis of the measured diffraction patterns was carried out using the EVA v.5.1.0.5 [66] program and the powder PDF-2 (Powder Diffraction File-2, ICDD, 2014) database [67].

#### 3.2.2. Determination of Microstructure Parameters from XRD Patterns by Methods of Analyzing the Profiles of Observed XRD Reflections

One of the main reasons for the broadening of XRD reflections is the broadening due to the small size of the crystallites and due to the presence of microstrains in the crystallites. To show the absence or presence of the contribution of crystallite microstrains to the observed broadening of reflections, WHP and SSP graphs were constructed using SizeCr v.11.01 program [37], which takes into consideration the type of the observed XRD reflections (pV type).

For calculations using the SizeCr program, it is necessary to know the parameters of independent X-ray reflections; therefore, strongly superimposed reflections that cannot be confidently separated due to the coincidence or closeness of their angular positions were not taken into account. The parameters of XRD reflections (their observed Bragg angles 2*θ*_B_^obs^, FWHMs *FWHM*, integral (*I*_int_) and maximum (*I*_max_) reflection intensities, and integral width *B*_int_ = *I*_int_/*I*_max_) were determined using a combination of TOPAS v.5.0 [68] and EVA [66] programs. To do this, first, the approximate positions of the observed reflections of the MBI phase were indicated in the TOPAS program, assuming a profile type adopted in TOPAS (for example, pV type). The reflection profiles were fitted by refining their angular positions 2*θ*_B_^obs^, *FWHM*, the parameters of the reflection profile, and the background parameters (for modeling the background and emission spectrum, see Section 3.2.5). Further, the fitted profiles of individual MBI reflections were extracted from the simulated XRD pattern and processed in the EVA program, which, after taking into account the background contribution and correcting the contribution of Cu-*K*_α2_ radiation, gave the desired reflection parameters.

The WHP and SSP graph points were calculated from the FWHMs of reflections corrected for the instrumental broadening (designated as *FWHM*_corr_) for pV reflections [69] observed in the measured XRD patterns. The values of the angular positions of the reflections with corrections for Δ2*θ*_zero_ and Δ2*θ*_displ_,

(4) $$2\theta_{B}=2\theta_{B}^{obs}+∆2\theta_{zero}+∆2\theta_{displ}\cdot \cos\left(\theta_{B}^{obs}\right),$$

were used for calculations. When constructing the WHP and SSP plots, we used the coefficient *K*_strain_ = 4 [70] of the Stokes equation, relating the broadening *FWHM*_strain_ due to the presence of microstrain *ε*_s_ with tg(*θ*_B_), and the Scherrer coefficient *K*_Scherrer_ = 0.94 [71] in the Scherrer equation, and relating the broadening *FWHM*_size_ due to the size *D* of crystallite with cos(*θ*_B_).

If there is a contribution of microstrains to the broadening of reflections, the average size *D* of nanocrystallites and the average value of microstrains *ε*_s_ in them is determined from the slope of the linear approximating lines *Y* = *A* + *B*·*X* of WHP or SSP and the value of their intersection with the *Y* axis of the graphs [37] (expressions for *X* and *Y* of WHP and SSP graphs for pV reflections are shown in the captions of corresponding axes of Figure 3a,b and in [37]).

For WHP method (pV reflections) [37]:

(5) $$D=\frac{K_{Scherrer}\cdot \lambda }{A},$$

(6) $$\varepsilon_{s}\left(\%\right)=B^{1/2}\cdot 100\%.$$

In case of SSP technique (pV reflections) [37]:

(7) $$D=\frac{1}{B\;},$$

(8) $$\varepsilon_{s}\left(\%\right)=\frac{2\cdot A}{K_{strain}}\cdot 100\%.$$

Both of the *A* and *B* values must be greater than zero in this case. In the case of *B* < 0 for WHP (Equation (6) or *A* < 0 for SSP (Equation (8)), it is assumed that the microstrain is zero (*ε*_s_ = 0) [36,37]). Correspondingly, for WHP and SSP, if *A* ≤ 0 in Equation (5) or *B* ≤ 0 in Equation (7), then *D* = ∞ (ideal perfect single crystal) [37].

In the absence of the contribution of microstrains to reflection broadening (model *ε*_s_ = 0), the crystallite sizes *D*_0_*^hkl^* were estimated for every individual reflection *hkl* using the Scherrer equation and averaged by the least squares method over the entire set of observed reflections.

#### 3.2.3. Rietveld Quantitative Analysis

Using the angular positions 2*θ_B_* of the extracted individual reflections (after correction for Δ2*θ*_zero_ and Δ2*θ*_displ_ according to (4)), the unit cell parameters of the crystalline phases recorded in the samples were calculated by the least squares method utilizing the program Celsiz v.1.1 [72]. The obtained values of the unit cell parameters were used as starting points in the Rietveld fitting [73] of the calculated XRD pattern to the experimental one using the TOPAS v.5.0 program [68]. The coordinates of the atoms of the structure of the MBI phases for the Rietveld analysis were taken from the Cambridge Crystallographic Data Center (CCDC) [74] (code 1199885). The same atomic coordinates were utilized for all MBI phases.

Quantitative Rietveld analysis was carried out. In the course of fitting the simulated XRD patterns to the experimental ones, the weight contents of crystalline phases in the sample were determined by the TOPAS program from refined scaling factors (scale-factors) according to the well-known formalism [75].

Of the structural parameters, only the parameters of the unit cell and the isotropic temperature factors *B*_iso_^overall^ of the atoms, which are common to all atoms in the structures of each phase, have been refined. The atomic coordinates were fixed and not refined for all phases during the quantitative Rietveld analysis.

When modeling calculated XRD patterns in the TOPAS program, the emission spectrum (Cu-*K_α_* doublet) was described by the Berger model of 5 spectral lines with different FWHMs [76]. The standard weight scheme *w_i_* = 1/*y_i_* was used, where *y_i_* is the intensity of the XRD pattern at the point 2*θ_i_* = 2*θ*_start_ + (*i* − 1)∙Δ2*θ*_step_. Atomic factors of neutral atoms were used.

The background was modeled using the Chebyshev polynomial of the 7th order and the contribution of a hyperbolic additive for 2*θ* < 10° range. The amorphous halo (for MBI-PG samples) was considered as an addition to the background and was modeled using the split Pearson VII function (SPVII) [68,77], which allows modeling asymmetric peaks.

The typical course of fitting was as follows. In the start of the Rietveld fitting, after setting the unit cell parameters of the observed crystalline phases to the start values determined in the calculations by Celsiz, the angular corrections Δ2*θ*_zero_ and *displ* were first refined,

(9) $$∆2\theta_{displ}=2\cdot \frac{displ}{R_{gon}}\cdot cos(\theta ),$$

where *R*_gon_ is the known radius of the diffractometer goniometer. Since the angular correction Δ2*θ*_zero_ was already introduced into the experimental XRD patterns from measurements of the mixture of the samples with the certified powder, the angular corrections obtained by Rietveld refinement were close to zero.

After that, the unit cell parameters were included in the refinement, but they were refined in different cycles with angular corrections to avoid correlations. The XRD patterns were fitted using instrumentally broadened XRD reflection profiles calculated from the geometry and diffractometer slits used (FP (first principles) profiles in the TOPAS program [68] and FP (fundamental parameters) profiles in [78]), and assuming that the broadening of reflections occurs due to the size of the crystallites and the contribution of microstrains to the broadening. This made it possible to refine the Gaussian and Lorentzian components of the mean crystallite size and microstrain in accordance with the double-Voigt approach [79] adopted in TOPAS, and to calculate their values.

In TOPAS, the crystallite size *D*^TOPAS^ is designated as *Lvol-FWHM*. To compare with the microstrain values *ε*_s_^WHP^ and *ε*_s_^SSP^ obtained, respectively, in WHP and SSP calculations done by program SizeCr, the microstrain parameter *e*_0_ obtained by TOPAS was recalculated to

(10) $$\varepsilon^{TOPAS}\left(\%\right)=2\cdot e_{0}\cdot 100\%$$

(see [61] for explanation).

In order to compare the obtained crystallite size *D*^TOPAS^ values with those (*D*^SSP^ and *D*^WHP^) estimated by the SizeCr program, the same Scherrer coefficient *K*_Scherrer_ = 0.94 was used in Rietveld refinement as for SizeCr. Initially, for all crystalline phases, microstrains were not included in the refinement. Based on the results obtained during the construction of WHP and SSP plots, at subsequent stages of refinement, microstrain was included in the refinement for the crystalline phase with largest *D*^TOPAS^ for which WHP and SSP plots showed non-zero microstrain values (for the phase with the smallest crystallite size *D*^TOPAS^ ~2.5 nm, for which no reflections were observed in the XRD patterns due to their wide FWHMs, the absence of microstrains was also assumed). As a rule, taking into account the contribution of the microstrain for the phase mentioned above gave an improvement in weighted profile factor *R*_wp_ of only ~0.05%.

The inclusion in the Rietveld refinement of the Gaussian and the Lorentzian component of the microstrain parameters for all phases, for which the absence of the contribution of microstrains to the broadening of the reflections was initially supposed, led to *ε*_s_^TOPAS^ ≈ 0 within e.s.d. and did not improve the quality of fitting of the XRD patterns, which confirmed the suppositions made about the absence of microstrains in these phases. An example of this inclusion of the microstrain in refinement for all phases is shown in Table 3 for MBI-ChA and MBI-ChA-mill samples.

The inclusion in the refinement of the parameters of the preferred orientation of crystallites according to the March-Dollase (MD) [80] model along the crystallographic directions, along which the reflections which showed the greatest intensity, gave a decrease in *R*_wp_ by ~1% for all Rietveld fittings done. The influence of other directions of predominant orientation was corrected using the 8th order spherical harmonics model [81], which led to a further decrease in *R*_wp_ by ~1.5%. Structural parameters and preferred orientation parameters were refined in different cycles to avoid correlations between them.

At the last stage, the structure parameter was refined, namely, the total isotropic temperature coefficient of atoms, which led to a further decrease in *R*_wp_, as a rule, by ~0.05%. Refinement of the background parameters and the scale factor was carried out at each stage of fitting. The procedure for refining the structural and non-structural parameters was repeated until their change stopped.

The quality of the fitting of the XRD patterns was controlled visually (graphically), as well as quantitatively using a weighted profile agreement factor *R*_wp_. Additionally, at each step, the decrease in other agreement factors, the profile *R*_p_, the weighted profile c*R*_wp_, and the profile c*R*_p_ corrected for the background contribution, as well as the Bragg factor *R*_B_, was checked (for the definition of agreement factors, see, for example [68,82]). Weighted profile factor c*R*_wp_ and profile factor c*R*_p_ are calculated using program RietEsd v.6.03 [83] due to wrong values given by TOPAS v.5.0, when using the hyperbolic additive to background (see explanations in [83]).

The factor *m*_e.s.d._, which corrects the e.s.d.s of the refined parameters, obtained in the Rietveld program, for serial correlation according to the procedure [84] by multiplication, was estimated using the RietEsd v.6.03 [83] program.

#### 3.2.4. Le Bail Fitting

The LB fitting [43], which does not require knowledge of atomic coordinates, but only approximate values of the unit cell parameters and the space group of symmetry of crystal phases, was carried out for the MBI-ChA and MBI-ChA-mill samples, characterized by the strongest influence of the effects of the preferential orientation. In the LB method, the intensity of reflections is not calculated on the basis of a structural model, but is extracted from the angular position and reflection profile calculated on the basis of refined unit cell parameters and refined profile parameters directly from the XRD pattern during fitting. As a result, the LB technique, even in the case of strong preferential orientation effects, allows you to get a good quality fitting of the simulated XRD pattern to the experimental one without using preferential orientation models.

The good quality of the LB fitting with a description of the profiles of all observed reflections indicate the presence of crystalline phases in the sample that were used for fitting. The LB method results in obtaining precise refined values of the parameters of the unit cells of the crystalline phases. When using the reflection profiles of the FP type, the LB method also gives, with good precision, the values of the parameters of the microstructure, mean crystallite size *D*^TOPAS^, and absolute value *ε*^TOPAS^ of the mean microstrain.

The course of the LB fitting and the list of refined structural and non-structural parameters are completely similar to those described in Section 3.2.3 for the Rietveld fitting, with the exception of parameters related to atoms (temperature factors of atoms, in particular) and with a preferential orientation model, which are not involved for the LB fitting.

Unlike the Rietveld method, the LB method does not allow for obtaining the weight content of the phases by refinement, since there is no information about the content of the unit cell. Nevertheless, taking into account that the molecular weights of different MBI-ChA phases are the same, the weight content of the phases was estimated as the ratio of the areas under the XRD reflections of the MBI-ChA_i_ (*i* = 1, 2) phase extracted from the simulated XRD pattern of this phase and the total area of reflections of both MBI phases (after subtraction of the background contribution),

(11) $${\;\;\;\;\;\;\;\;\;\;Wt}_{i}=\frac{\sum I_{int}^{{MBI-ChA}_{i}}}{\sum I_{int}^{calc}},$$

where $I_{int}^{{MBI-ChA}_{i}}$ is the integral intensity of reflections of the MBI-ChA*_i_* phase, and $I_{int}^{calc}$ is the total integral intensity of reflections of both phases, MBI-ChA_1_ and MBI-ChA_2_.

#### 3.2.5. Calculation of Correlation Function

To analyze the amorphous-like XRD pattern of MBI-MS, the method of total correlation function *c*(*r*) was chosen [84]. The construction of this function from the measured XRD pattern is described in more detail in Section S2 of Supplementary Materials.

Briefly, in this method, the experimental XRD pattern *I*(2*θ*) is first corrected for the contributions of Cu-*K_α_*_2_ radiation (for example, using the EVA program) and a small-angle background (utilizing a graphical program, for example, Origin v. 6.0 or later [85]). After that, *I*(2*θ*) is also corrected for Lorentz, polarization, and absorption factors (for formulas, see, for example, [68,86,87]) and normalized to the function $<f_{corr}^{2}>$ at the high diffraction angles 2*θ* > 90°,

(12) $$<f_{corr}^{2}>=\sum ({c_{i}{\cdot f}_{i}^{corr})}^{2},$$

where *c_i_* is the atomic concentration of element *i* in the sample, and *f_i_*^corr^ is the atomic scattering factor *f_i_* of element *i*, calculated analytically [88] and corrected for the coefficients Δ*f_i_*′ and Δ*f_i_*″ of anomalous dispersion.

By Fourier transformation of the function (*S*(*Q*) − 1), a correlation function *c*(*r*) is obtained,

(13) $$c\left(r\right)=2\cdot \frac{r}{\pi }\int Q\cdot \left(S\left(Q\right)-1\right)\cdot \sin\left(Q\cdot r\right)dQ,$$

where *r* is a correlation distance, *S*(*Q*) is the total structure factor of the sample,

(14) $$S\left(Q\right)=\frac{I_{corr}^{norm}-<f_{corr}^{2}>+{<f_{corr}>}^{2}}{{<f_{corr}>}^{2}},$$

$I_{corr}^{norm}$ is the XRD pattern after all corrections and normalization,

(15) $${<f_{corr}>}^{2}=(\sum {c_{i}\cdot f_{i}^{corr})}^{2},$$

and all the dependences on the diffraction angle 2*θ* are recalculated depending on the modulus of the scattering wave vector;

(16) $$Q=4\pi \cdot \frac{sin(\theta )}{\lambda }$$

where λ is the wavelength of Cu-*K_α_*_1_ radiation (after correction of the Cu-*K_α_*_2_ contribution), *θ* is half the diffraction angle.

The correlation function *c*(*r*) does not require information about the average atomic density of the sample for its construction. The maxima of *c*(*r*) correspond to the mean interatomic distances in the sample (with the exception of “false” maxima arising from the breakage of the Fourier series, which usually appear at small values of the correlation distance *r*).

### 3.3. Optical and Dielectric Measurements

Measurements of the IR absorption spectra (Fourier-transform infrared (FTIR) spectra) were carried out using an IR-Fourier spectrophotometer IRPrestige-21 (Shimadzu Corporation, Kyoto, Japan) with an IR microscope AIM-8000 (Shimadzu Corporation, Kyoto, Japan), both in the specular reflection mode and in the transmission mode, followed by the Kramers–Kronig transformation. The results were then converted to absorbance. The measured spectral range was from 650 to 5000 cm^−1^. Measurements were carried out on split asbestos fibers shown in Figure 16f.

The photoluminescence spectrum was measured using diffraction spectrometers DFS-24 (LOMO, Saint Petersburg, Russia) and FSD-8 (Optofiber LLC, Moscow, Russia) with 1200 lines/mm and 300 lines/mm gratings, respectively. PMT and CCD matrix were used for radiation detection. Luminescence was excited by CW radiation of a laser operating at a wavelength of 405 nm.

Measurements of capacity and dielectric losses in samples of porous borate glasses and asbestos with and without MBI were performed in the frequency range of 25 Hz^−1^ MHz with LCR-meters MIT 9216A (Protek, Seattle, WA, USA) and E7-20 (MNIPI, Minsk, Belarus), using the LabView software package (Version 2011, NIST, Gaithersburg, MD, USA). Thin metallic foil was used as electrodes for measurements of capacity and dielectric losses of samples.

## 4. Conclusions

This study showed that MBI molecules from a melt, gas phase, or solution of MBI easily penetrate into the nanopores of borate glasses, nanotubes of chrysotile asbestos, and mesoporous silica without the use of external pressure, which makes it possible to almost completely fill the PG and ChA pores from the melt or from the gas phase. A lower degree of filling is realized in MS when the pores are filled with an MBI solution in alcohol.

According to our XRD measurements, the incorporation of MBI into PG with pore sizes of *D*_pore_ ~ 2.5 and 7 nm is accompanied by the appearance in the matrix of various phases which have the structure of an MBI single crystal with slightly different crystal parameters, paramters *a* and *c*. The sizes of the crystallites of some phases can significantly exceed the sizes of PG pores. The appearance of such crystallites indicates the correlation of the crystal lattice in the pores, and the correlation diameter can significantly exceed *D*_pore_. In MBI-PG2.5, there are two such phases with sizes of *D* ~ 100 nm and 15 nm, while, in MBI-PG7, there are three phases, with *D* ~ 60, 30, and 18 nm. In both MBI-PG samples, there is also a phase with crystallite sizes that are equal to or smaller than the pore sizes. The volumes occupied by the phase *V_i_* are approximately inversely proportional to the sizes of crystallites *D_i_*, and the number of crystallites *N_i_* ~*D_i_^−4^*. Thus, the maximum volume of MBI-PG is occupied by the phase with *D* ≤ *D*_pore_, and much smaller volumes are occupied by phases with *D* > *D*_pore_. In MBI-ChA, two phases are observed with very similar crystal parameters, parameters *a* and *c*, which occupy approximately the same volumes. The characteristic size of the crystallites of these phases approximately coincides with the diameter of the nanotubes. This suggests that there is no correlation of crystallites in different ChA nanotubes.

In MBI-MS, the XRD patterns do not show reflections which correspond to the MBI lattice, since the degree of filling of the pores is much less than in the MBI-PG and MBI-ChA samples and there is no correlation of the crystal structure, even if it exists. As a result, the width of the reflections must be very large. The presence of MBI in MS, however, manifests itself in the features of the correlation functions, *c*(*r*).

The presence of MBI molecules in the studied structures was confirmed in optical experiments. In particular, the bands corresponding to the molecular vibrations of MBI are well manifested in the IR spectra of the MBI–ChA. In all of the studied structures, MBI-PG, MBI-ChA, and MBI-MS, the introduction of MBI is accompanied by the appearance of strong photoluminescence. The spectral dependence of PL in the MBI-PG2.5 and MBI-ChA samples is similar to the spectrum of the MBI single crystal; however, the observed bands are broadened and slightly shifted to the short wavelength region, which may be due to the manifestation of the nanoparticle size effect. The MBI-PG7 and MBI-PG30 “blue sifts” are much smaller, since the size of the nanoparticles approaches those that are typical for bulk crystals. The widest and most weakly structured PL spectrum is observed in MBI-MS. From a practical point of view, the bright luminescence of these nanostructures makes them promising materials for application as medical biosensors.

The introduction of MBI into PG and ChA is accompanied by a change in the dielectric properties of the matrices. A strong decrease in the effective dielectric permittivity *ε*_eff_ and dielectric loss *tgδ* at low frequencies is associated with the substitution of MBI molecules in pores and nanotube matrices, for example, water adsorbed from air. Our calculations show that the value of *ε*_eff_ in MBI-PG structures is mainly determined by the total pore volume filled with MBI and weakly depends on the pore size. The conductivity *σ* of PG, ChA, MBI- PG, and MBI-ChA at high frequencies *ω* is frequency dependent and is described by a power dependence *σ* ~ *ω ^s^*, which indicates a hopping type of conduction. The main parameters of hopping conductivity—energy barrier, *W_m_*, and the carrier localization radius *a* in structures with pore sizes *D*_pore_ ≥ 7 nm, are close in value. In contrast, in the MBI-PG2.5 structure, with a smaller pore size, *D*_pore_ ~2.5 nm, a significantly smaller value of *W_m_* and a larger value of activation energy *E_a_* for DC conductivity are observed, which indicates the manifestation of a nanoparticle size effect.

## Figures and Tables

**Table 1:** Results of Rietveld quantitative analysis of the sample MBI-PG2.5 obtained by means of Rietveld program TOPAS (unit cell parameters a and c and volume *V*_cell_, size *D*^TOPAS^ of nanocrystallites and microstrain *ε*_s_^TOPAS^, weight content of the crystalline phases *Wt*, overall isotropic temperature factor of atoms *B*_iso_^overall^, March-Dollase parameter *r*_MD_ of the preferential orientation), and fitting quality characteristics (agreement Bragg factor *R*_B_, weighted profile factor *R*_wp_ and profile factor *R*_p_, weighted profile factor c*R*_wp_ and profile factor *cR_p_* corrected to background contribution). Additionally, microstructure parameters *D*^SSP^ and *ε*_s_^SSP^ estimated using SSP method and factors *m*_e.s.d._ for correction of e.s.d.s, obtained in Rietveld refinement, are shown.

Crystalline Phase ^a^	*a*, Å *c*, Å	*V*_cell_, Å^3^	*D*^TOPAS^, nm *ε*_s_^TOPAS^, %	*D*^SSP^, nm *ε*_s_^SSP^, %	*Wt*, wt.% *B*_iso_^overall^, Å^−2^	*R*_B_, % *r*_MD_ ^b^	*R*_wp_, % *R*_p_, %	c*R*_wp_, % c*R*_p_, %
MBI-PG2.5_L_ + MBI-PG2.5_M_ + MBI-PG2.5_S_, *m*_e.s.d._ = 7.529 ^c^								
MBI-PG2.5_L_	13.979(8)	1220.3 (2.1)	107(1)	119(20) ^d^	0.80(2)	2.68	1.36 1.00 7.529	18.86 19.86
	7.211(11)		0.082(1)	0.16(4) ^d^	0.5(5)	0.34(1)		
MBI-PG2.5_M_	13.873(60)	1209.4 (7.5)	12(1)	15(5) ^d^	25.5(7)	0.39		
	7.256(9)		0	0 ^d^	4.3(4)	0.53(1)		
MBI-PG72.5_S_	14.060(45)	1240.0 (14.0)	2.4(2)	–	73.7(7)	1.07		
	7.243(75)		0	–	*B*_iso_^overall^_MBI-M_	5.8(6)		

^a^ space group of the MBI phases is *P*4_2_/*n* (86) (Choice 2 according to [38]). Table unit cell parameters according to Cambridge Crystallographic Data Center (CCDC) databank, *a* = 13.950(9) Å, *c* = 7.192(3) Å, and unit cell volume *V*_cell_ = 1212.1(1.2) Å^3^ (CCDC code 1199885). ^b^ consistent with [110], [001], and [211] for MBI-PG2.5_L_, MBI-PG2.5_M_, and MBI-PG2.5_S_, respectively. ^c^ all e.s.d.s of the parameters shown in the table and obtained by refinement during Rietveld fitting are corrected on underestimation due to serial correlations by multiplication on the coefficient *m*_e.s.d_. ^d^ WHP calculations result in close values, *D*^WHP^ = 100(36) nm and *ε*_s_^WHP^ = 0.13(6)% for MBI-PG2.5_L_, *D*^WHP^ = 15(5) nm, and *ε*_s_^WHP^ = 0 for MBI-PG2.5_M_ phase.

**Table 2:** Results of Rietveld quantitative analysis of the sample MBI-PG7 obtained by means of Rietveld program TOPAS (see head of Table 1 for description of parameters shown).

Crystalline Phase ^a^	*a*, Å *c*, Å	*V*_cell_, Å^3^	*D*^TOPAS^, nm *ε*_s_^TOPAS^, %	*D*^SSP^, nm *ε*_s_^SSP^, %	*Wt*, wt.% *B*_iso_^overall^, Å^−2^	*R*_B_, % *r*_MD_ ^b^	*R*_wp_, % *R*_p_, %	c*R*_wp_, % c*R*_p_, %
	MBI-PG7_L_ + MBI-PG7_M1_ + MBI-PG7_M2_ + MBI-PG7_S_, *m_e.s.d._* = 5.832 ^c^							
MBI-PG7_L_	13.943(2)	1224.3 (6)	61(3)	57(5) ^d^	0.80(2)	0.47	1.63 1.27	13.02 16.15
	7.272(3)		0.014(6)	0.06(12) ^d^	1.0(4)	1.24(3)		
MBI-PG7_M1_	14.025(11)	1230.1 (1.4)	28(1)	31(3) ^d^	5.70(9)	0.40		
	7.221(2)		0	0 d	4.3(6)	0.62(1)		
MBI-PG7_M2_	14.014(17)	1222.9 (2.9)	17(1)	18(4) ^d^	9.71(17)	1.26		
	7.190(12)		0	0 d	3.9(5)	0.21(1)		
MBI-PG7_S_	13.910(58)	1202.5 (9.1)	2.7(1)	–	71.33(14)	0.27		
	7.176(34)		0	–	*B*_iso_^overall^_MBI-M2_	0.10(1)		

^a^ see corresponding note to Table 1. ^b^ corresponds to [001], [012], [241], and [211] for MBI-PG7_L_, MBI-PG7_M1_, MBI-PG7_M2_, and MBI-PG7_S_, respectively. ^c^ see corresponding note to Table 1. ^d^ WHP calculations result in close values, *D*^WHP^ = 56(7) nm and *ε_s_*^WHP^ = 0.10(9)% for MBI-PG7_L_, *D*^WHP^ = 31(3) nm and *ε*_s_^WHP^ = 0 for MBI-PG7_M1_, and *D*^WHP^ = 18(4) nm and *ε*_s_^WHP^ = 0 for MBI-PG7_M2_.

**Table 3:** Results of LB fitting of the XRD patterns of MBI-ChA and MBI-ChA-mill samples obtained by means of Rietveld program TOPAS (see head of Table 1 for description of parameters shown).

Crystalline Phase ^a^	*a*, Å *c*, Å	*V*_cell_, Å^3^	*D*^TOPAS^, nm ^b^ *ε*_s_^TOPAS^, % ^b^	*Wt*, wt.% ^c^	*R*_wp_, % *R*_p_, %	c*R*_wp_, % c*R*_p_, %
MBI-ChA, *m*_e.s.d._ = 4.715 ^d^						
MBI-ChA_1_	14.062(1) 7.156(1)	1225.4(2)	9.98(6) 0.000(3)	62.4	3.58 2.49	4.01 2.91
MBI-ChA_2_	13.630(3) 7.129(2)	1147.0(5)	9.13(7) 0.006(7)	35.6		
MBI-ChA-mill, *m*_e.s.d._ = 7.492 ^d^						
MBI-ChA_1_	14.063(3) 7.160(4)	1226.3(8)	9.41(7) 0.000(70)	77.3	3.87 2.33	4.71 3.12
MBI-ChA_2_	13.620(6) 7.131(2)	1145.6(8)	8.43(11) 0.002(90)	22.7		

^a^ Space group of the MBI phases is *P*4_2_/*n* (86) (Choice 2 according to [38]). Table unit cell parameters according to CCDC databank, *a* = 13.950(9) Å, *c* = 7.192(3) Å, and unit cell volume *V*_cell_ = 1212.1(1.2) Å^3^ (CCDC code 1199885). ^b^ Both WHP and SSP calculations result in the same values of *D* = 8.0(2.5) nm and *ε*_s_ = 0 for MBI-ChA. ^c^ The *Wt* values are estimated by the relative areas under the XRD reflections of the MBI-ChA_1_ and MBI-ChA_2_ phases on the XRD pattern, taking into account the same molecular weights of the MBI1 and MBI2 phases (see Section 3.2.4). The mean weight contents of the MBI-ChA_1_ and MBI-ChA_2_ phases averaged over MBI-ChA and MBI-ChA-mill samples are *Wt* = 70(7) wt.% and 30(7) wt.%, respectively. ^d^ All e.s.d.s obtained during LB refinement are corrected for underestimation due to serial correlations by multiplying by a coefficient *m*_e.s.d_.

**Table 4:** Wavenumber (*ν*) positions of the absorption peaks in the IR spectra of an asbestos sample with the addition of MBI. The last column shows the interpretation of lines for MBI from [46]. Abbreviations: *Γ*, out-of-plane bending; δ, planar bending; ν, stretching (stretching vibrations); M, methyl group.

*ν*, cm^−1^	Assignment from [46]
1219	δ_CCH_ + ν_CN_ + ν_CC_
1271	ν_CN_ + ν_CC_ + δ_CCH_
1360	ν_CCH_ + ν_CN_
1389	ν_CC_ + ν_CN_ + Mδ_CH2_
1418	Mδ_CH2_ + δ_CCH_
1448	δ_CCH_ + ν_CC_
1485	Mδ_CH2_ + *Γ*_CCCN_ + δ_CCH_
1555	ν_CN_ + ν_CC_
1624	ν_CC_ + ν_CN_
2544	
2583	
2679	
2725	
2756	
2791	
2847	
2876	
2918	
2995	
3061	Mν_CH_
3096	Mν_CH_
3111	Mν_CH_
3177	ν_CH_

**Table 5:** Exponent *s* of conductivity frequency dependence and parameters characterizing hopping conductivity: energy barrier, *W_m_*; localization radius *a* of carriers; and activation energy *E_a_* in ChA, MBI-ChA, PG, and MBI-PG structures.

	*s*	*W_m_*, eV	*a*, Å
ChA	0.80	0.77	12
MBI-ChA	0.94	2.58	4.4
MBI-PG30	0.91	1.72	6.6
PG7	0.90	1.55	5.1
MBI-PG7	0.94	2.58	7.3
MBI-PG2.5	0.79	0.74	18.1

## Data Availability

The data presented in this study are available on request from the corresponding author.

## Supplementary Materials

The following supporting information can be downloaded at: https://www.mdpi.com/article/10.3390/ijms241813740/s1.

## Abbreviations

MBI	2-methylbenzimidazole or C_8_H_8_N_2_
*D*_pore_	characteristic pore or tube diameter
*d*_mol_	maximum molecular size of MBI
*D*	characteristic size of crystallites
*a*	MBI lattice parameter
*c*	MBI lattice parameter
*V*_cell_	unit cell volume
PG	borate porous glasses
ChA	chrysotile asbestos
MS	mesoporous silica
PG2.5	PG with a pore diameter of 2.5 nm
PG7	PG with a pore diameter of 2.5 nm and 7 nm
MBI-PG2.5	PG2.5 filled by MBI from melt
MBI-PG7	PG7 filled by MBI from melt
MBI-PG7g	PG7 filled by MBI from gas
MBI-MS	mesoporous silica filled by MBI
PL	photoluminescence
Phases in MBI PG2.5	
MBI-PG2.5_L_	MBI phase Large
MBI-PG2.5_M_	MBI phase Mean
MBI-PG2.5_S_	MBI phase Small
Phases in MBI-PG7	
MBI-PG7_L_	MBI phase Large
MBI-PG7_M1_	MBI phase Mean1
MBI-PG27_M2_	MBI phase Mean2
MBI-PG7_S_	MBI phase Small
Phases in MBI-ChA	
MBI-ChA_1_	MBI phase 1
MBI-ChA_2_	MBI phase 2
*E*_ph_	photon energy
*f*, ω	frequency, *ω* = 2π*f*
*ε*_eff_	effective permittivity
*tgδ*	dielectric loss
*σ*	conductivity
