# Supplementary material for: Synthesis and Study of Organic Nanostructures Fabricated by Inclusion of 2-Methylbenzimidazole Molecules in Nanotubes of Chrysotile Asbestos, Mesoporous Silica, and Nanopores of Borate Glasses
Source: Int J Mol Sci. 2023 Sep 6;24(18):13740. doi: 10.3390/ijms241813740 (PMC10531229; doi:10.3390/ijms241813740)
Supplement: Supplementary file 1 [file ijms-24-13740-s001.zip › ijms-2587074-supplementary.pdf]

Supplementary Materials

Synthesis and study of organic nanostructures fabricated by inclusion of 2-methylbenzimidazole molecules in nanotubes of chrysotile asbestos, mesoporous silica, and nanopores of borate glasses.

Elena Balashova[1*], Aleksandr A. Levin[1], Sergey Pavlov[1], Anatoly Starukhin[1], Alexander Fokin[1], Dmitry Kurdyukov[1], Daniil Eurov[1], Boris Krichevtsov[1]

Ioffe Institute, Politechnicheskaya 26, 194021 Saint Petersburg, Russia
* Correspondence: balashova@mail.ioffe.ru

S.1. XRD results

S.1.1. Simulated XRD pattern of bulk MBI compound

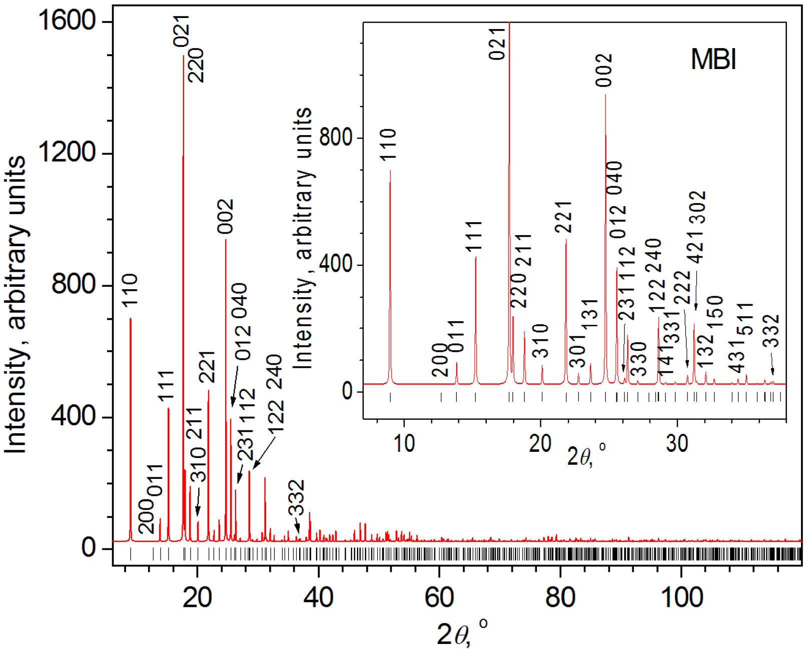

Figure S1. XRD pattern of bulk MBI compound simulated using structure data of CDCC databank (code 1199885). Miller indices *hkl* of selected reflections are indicated. In the case of highly overlapping reflections, the *hkl* of the reflection giving the greatest contribution is displayed. The insert shows a range of 2θ from 6° to 38°.

S.1.2. XRD results for MBI-PG7

In the case of the MBI-PG7 sample, the distribution of the individual D_0^{hkl} sizes of crystallites over the Bragg angles of the MBI reflections obtained in the zero microstrain model (ε_s = 0) does not show such an obvious division into separate ranges with the strongly different sizes of crystallites, but rather distributed as a "cloud" with a wide spread around a certain average value. However, even in this case, it is possible to divide

this "cloud" into different ranges with crystallite sizes D_0^{hkl} distributed around the average values with a smaller spread. Unlike the MBI-PG2.5 sample with a smaller pore size of ~2.5 nm, for the MBI-PG7 sample with a pore size of ~7 nm, it is already possible to distinguish not two, but three characteristic average sizes of crystallites corresponding to three MBI phases (Figure S2).

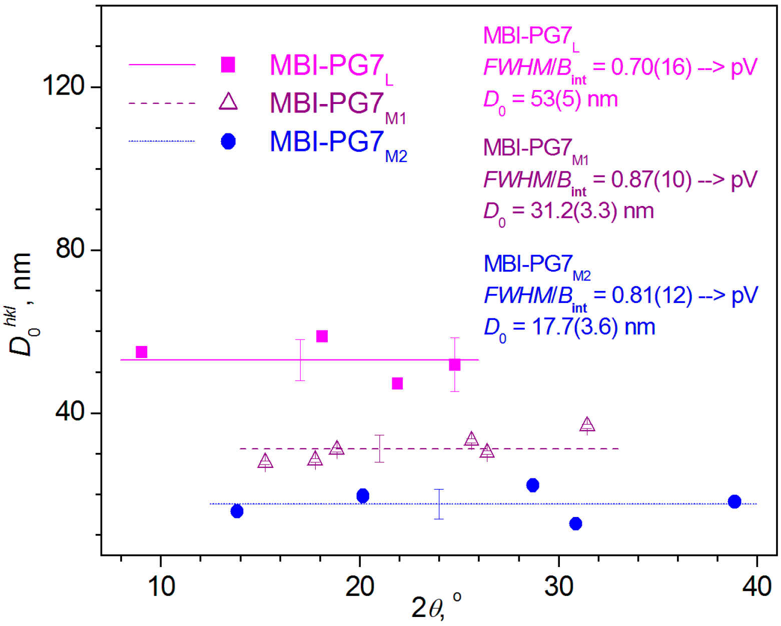

Figure S2. MBI–PG7 sample. 2θ angle distribution of crystallite sizes D_0^{hkl} of MBI-PG7$_L$, MBI-PG2.5$_{M1}$, and MBI-PG2.5$_{M2}$ phases calculated in the model of zero microstrain (ε_s = 0) for observed individual reflections of the phases with Bragg angle $2\theta = 2\theta_B$ (Bragg angle $2\theta_B$ is corrected to zero shift and displacement (Section 3.2.2 in the main text)).

Similar to the MBI-PG2.5$_L$ phase with a larger average crystallite size in the MBI-PG2.5 sample, for the MBI-PG7$_L$ phase of the MBI-PG7 sample, characterized by a larger value of the average crystallite size (D_0 = 53(5) nm), the experimental individual values of D_0^{hkl} lie somewhat better on a straight line with a negative slope (although not as pronounced as for MBI-PG2.5, cf. Figures 2 of main text and Figure S2), which indicates the possibility of nonzero microstrain in the crystallites of this phase. The other two phases, MBI-PG7$_{M1}$ and MBI-PG2.5$_{M2}$, are characterized by smaller ("mean") crystallite sizes D_0 = 31(3) nm and 18(4) nm, respectively, and show no signs of microstrain.

Really, in MBI-PG7, as in the case of MBI-PG2.5 sample, the WHP and SSP plots can be described by a linear function with a small spread of experimental points, assuming the presence of three MBI phases with different crystallite sizes (Table 2 of main text Figure S3).

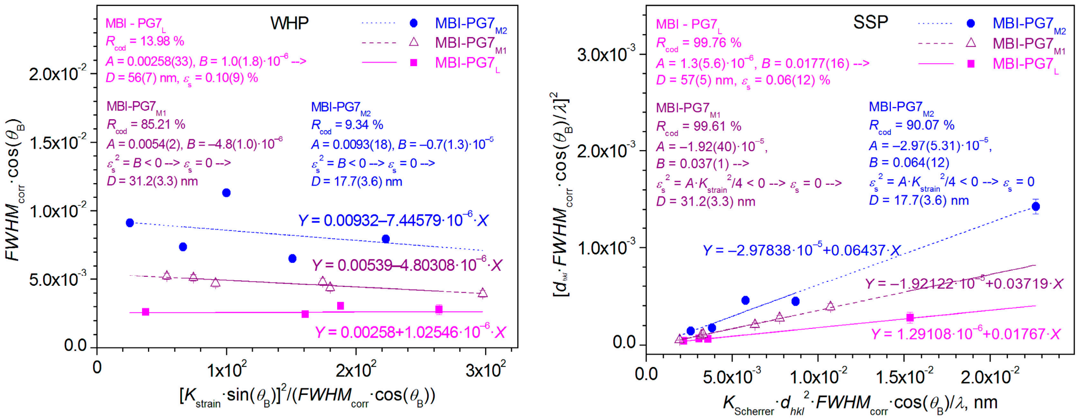

(**a**) (**b**)

Figure S3. (**a**) WHP and (**b**) SSP graphs, constructed for the reflections of MBI-PG7L, MBI-PG7M1, and MBI-PG7M2 crystalline phases of the MBI-PG7 sample. The designations are the same as shown in the caption to Figure 3 of the main text.

So, like for MBI-PG2.5 sample, both methods of profile analysis of XRD reflections of the MBI-PG7 sample, WHP and SSP, gave the same values of microstructure parameters D and ε_s within the e.s.d.s. As anticipated, the SSP chart is characterized by a much smaller spread of points around the straight plot, which is expressed by the higher value of the coefficient R_{cod} = 90.07–99.76 % compared to R_{cod} = 9.34–85.21 % for WHP (cf. Figures S3b and S3a), and, correspondingly, smaller e.s.d.s in case of SSP method, as a rule (Table 2 of main text).

As expected, in the MBI-PG7L like in MBI-PG2.5L phase, the presence of microstrain was found (ε_s^{SSP} = 0.06(12) % in comparison to ε_s^{SSP} = 0.16(4) % in MBI-PG2.5L). The crystallite size of the MBI-PG7L phase (D^{SSP} = 57(5) nm) is approximately two times smaller than that of the MBI-PG2.5L phase (D^{SSP} = 119(20) nm). The e.s.d. of microstrain is large, however, both methods, WHP and SSP, confidently and consistently show the possible presence of nonzero microstains in the crystallites of the MBIL phase. In the MBI-PG7M1 and MBI-PG7M2 phases, no microstrains are observed (ε_s^{SSP} = 0), as in the case of the MBI-PG2.5M phase of the MBI-PG2.5 sample. The average size of the crystallites of the MBI-PG7M1 phase is also about twice that in the case of MBI-PG2.5M, while the MBI-PG7M2 crystallites are approximately of the same size within the e.s.d. (D^{SSP} = 31(3) nm and 18(4) nm for the MBI-PG7M1 and MBI-PG7M2 phases of the MBI-PG7 sample, respectively, compared to D^{SSP} = 15(5) of the MBI-PG2.5M crystallites of the MBI-PG2.5 sample).

The results of quantitative Rietveld analysis confirmed and expanded the results of the X-ray line profile analysis. The Rietveld fitting, carried out under the assumption of only one MBI phase or two MBI phases with different crystallite sizes (MBI-PG7L and MBI-PG7M1 with crystallite sizes ~60 nm and ~30 nm, correspondingly), did not allow us to satisfactorily describe all the observed MBI reflections (achieved weighted profile agreement factor R_{wp} = 4.61% and 2.87%, respectively, but its analogue after subtracting the background contribution cR_{wp} = 65.50% and 38.70%). The inclusion of the parameters of the third phase MBI-PG7M2 with smaller crystallite size ~15 nm in the fitting led to a decrease in the weighted profile agreement factor to R_{wp} = 2.53%. Visually, the profiles of all the observed reflections were described quite satisfactorily. However, the factor cR_{wp} = 30.40% remained unsatisfactorily large. The assumption of the presence of the MBI-PG7S phase with even smaller crystallite sizes equal to or smaller than the pore sizes in the MBI-PG7 sample led to a

significant decrease in the agreement factors to R_{wp} = 1.64% and cR_{wp} = 13.02%, indicating on the presence of the MPB-PG7s phase.

S.1.3. XRD results for MBI-ChA and MBI-ChA-mill

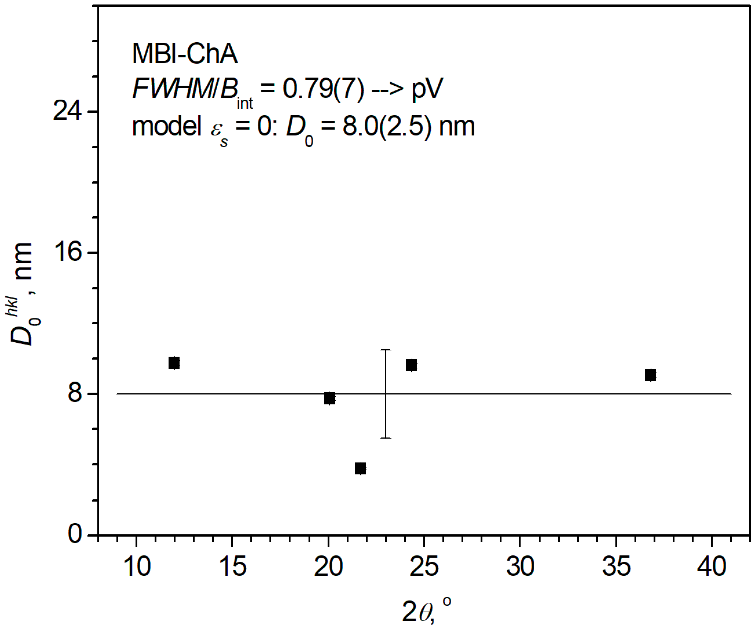

Figure S4. MBI-ChA sample. 2θ angle distribution of crystallite sizes D_0^{hkl} calculated in the model of zero microstrain (ε_s = 0) for observed individual MBI reflections. Only single non-overlapping reflections were taken into concidration, as well as individual reflections extracted from the general profile of observed overlapped reflections and reflections that make the greatest contribution to the observed general profile, if the contribution of other reflection is negligible.

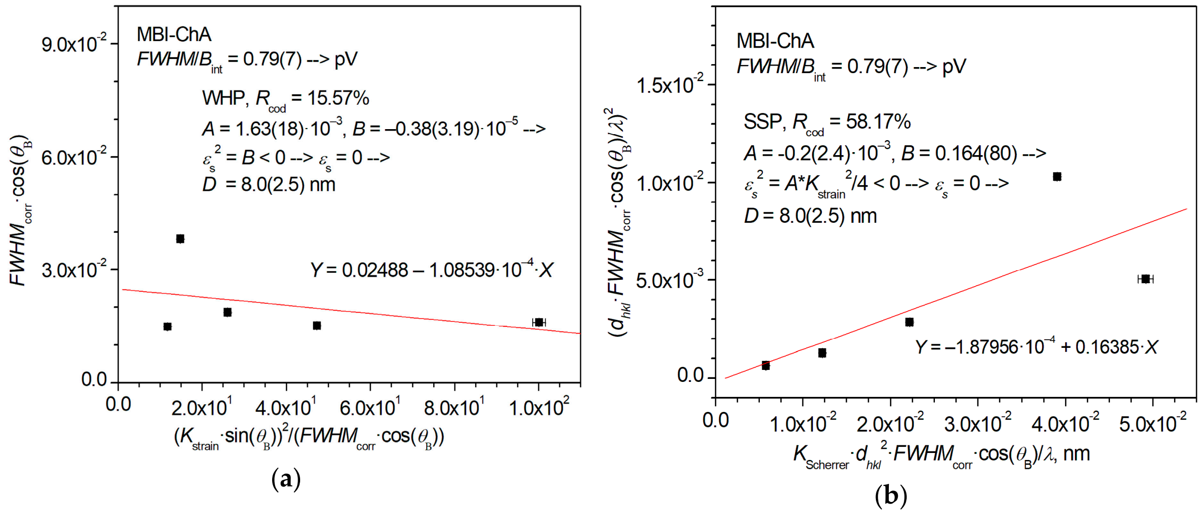

(**a**)

(**b**)

Figure S5. (**a**) WHP and (**b**) SSP graphs, constructed for the reflections of MBI-ChA sample. The designations are the same as shown in the caption to Figure 3 of the main text.

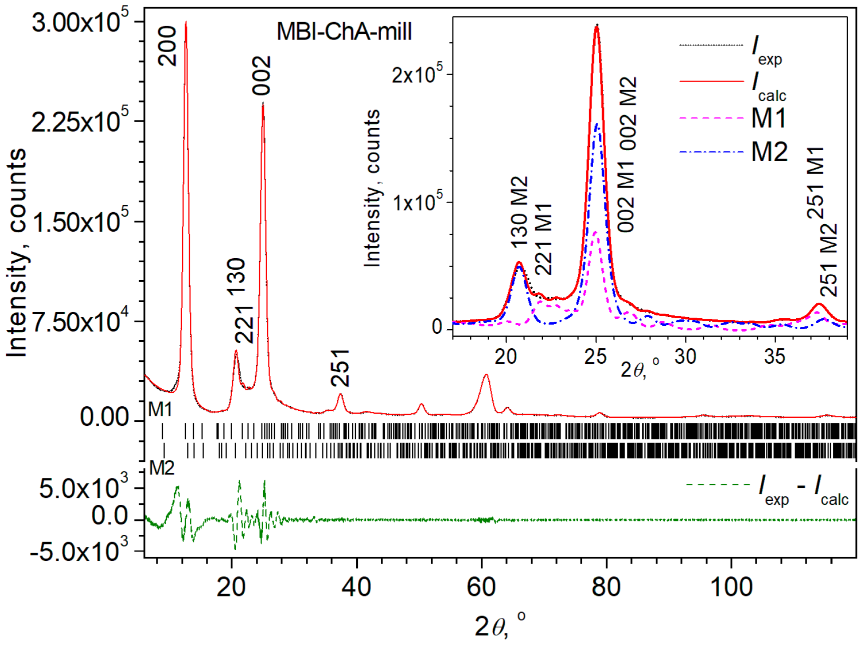

Figure S6. Sample MBI-ChA-mill. Results of the LB fit in frames of the two-phase MBI-ChA1 + MBI-ChA2 model. The MBI-ChA1 and MBI-ChA2 phases are designated as M1 and M2, respectively. Other details are the same as in caption to Figure 4 of main text. The positions of the phase reflections calculated from the unit cell parameters refined by the LB method are shown as vertical bars below. The experimental, calculated, and difference diagrams are marked as I_{exp}, I_{calc}, and $I_{exp} - I_{calc}$, respectively. The Miller indices *hkl* of some selected observed reflections are indicated. In the case of highly overlapping reflections, the *hkl* of the reflection giving the greatest contribution is displayed. The inset shows the experimental and calculated XRD patterns and calculated diagrams (together with the background contribution) corresponding to individual crystalline phases in the region $2\theta = 17°–39°$.

S.1.4. XRD results for MBI-MS and pure MS

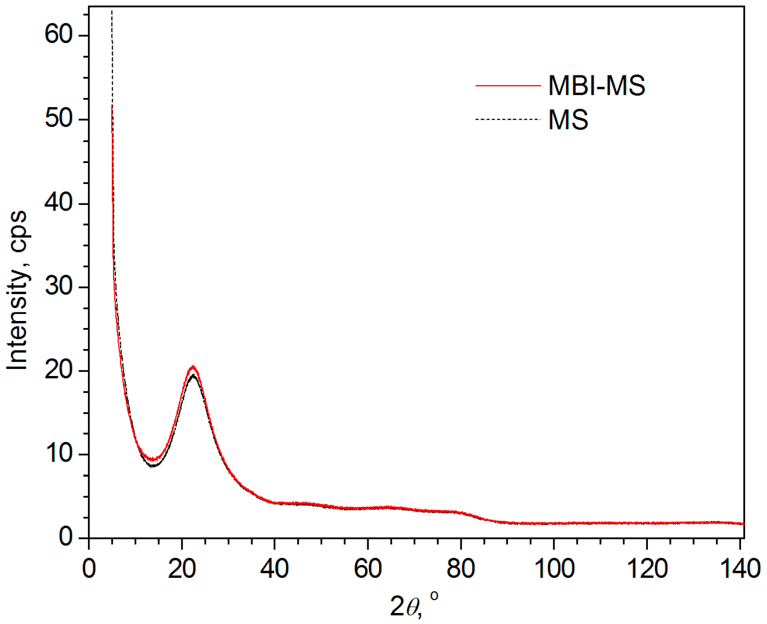

Figure S7. XRD patterns of the samples MBI-MS and pure MS.

S2. Calculation of correlation function

To analyze the amorphous-like XRD pattern of MBI-MS, the method of total correlation function $c(r)$ was chosen [1].

At the first step, the XRD pattern $I(2\theta)$ was corrected for the contribution of Cu-$K_{\alpha 2}$ radiation ($I_{corr1} = I - I_{Cu\text{-}K\alpha 2}$), for example, using the *EVA* [2] program. Then, using a graphical program, for example, Origin [3], background correction ($I_{corr2} = I_{corr1} - I_{bkgr}$) was performed by subtracting the background of the exponential decay function type to correct the small-angle region $I(2\theta)$. According to the well-known formulas (see, for example, [4, 5, 6], the intensities were corrected for Lorentz factors L, polarization P and absorption A,

$$I_{\text{corr}} = \frac{I_{\text{corr2}}}{LPA} \quad (1)$$

The resulting corrected function Icorr was normalized at the high angles $2\theta > 90°$ to the $<f_{corr}^2>$ function by multiplying by the scaling factor so that their sufficiently good coincidence was observed in the area of the high angles. Here

$$< f_{\text{corr}}^2 >= \sum(c_i \cdot f_i^{\text{corr}})^2, \quad (2)$$

where c_i is the atomic concentration of atomic element of sort i in the sample, and f_i^{corr} is the atomic scattering factor f_i of element i, calculated analytically [7] and corrected for coefficients of anomalous dispersion ($\Delta f_i'$ and $\Delta f_i''$) as

$$f_i^{\text{corr}} = ((f_i + \Delta f_i')^2 + (\Delta f_i'')^2)^{1/2}. \quad (3)$$

An interference function $Int(Q)$ was constructed from the resulting corrected normalized function $I_{\text{corr}}^{\text{norm}}$,

$$Int(Q) = \frac{I_{\text{corr}}^{\text{norm}} - <f_{\text{corr}}^2>}{<f_{\text{corr}}>^2}, \quad (4)$$

where

$$< f_{\text{corr}} >^2 = (\sum c_i \cdot f_i^{\text{corr}})^2, \quad (5)$$

and all the dependences on the diffraction angle 2θ are recalculated depending on the modulus of the scattering wave vector

$$Q = 4\pi \cdot \frac{\sin(\theta)}{\lambda}, \quad (6)$$

λ is the wavelength of Cu-$K_{\alpha 1}$ radiation (after correction of the Cu-$K_{\alpha 2}$ contribution), θ is half the diffraction angle.

Let note that $S(Q) = Int(Q) + 1$ is the total structure factor of the sample. By Fourier transformation of the function ($S(Q)$ - 1), a correlation function $c(r)$ is obtained,

$$c(r) = 2 \cdot \frac{r}{\pi} \int Q \cdot (S(Q) - 1) \cdot \sin(Q \cdot r)\, dQ, \quad (7)$$

where r is the correlation distance.

The correlation function $c(r)$ does not require information about the average atomic density of the sample for its construction. The maxima of $c(r)$ correspond to the mean interatomic distances in the sample (with the

exception of "false" maxima arising from the breakage of the Fourier series, which usually appear at small values of the correlation distance *r*).

References

1. Paufler, P.; Filatov, S.K.; Shakhverdova, I.P.; Bubnova, R.S.; Reibold, Müller, B.; Levin, A.A.; Meyer, D.C. Mechanical properties and structure of a nanoporous sodium borosilicate glass. *Glass Phys. Chem.* **2007**, *33*, 187–198. https://doi.org/10.1134/S1087659607030017
2. Bruker AXS. *Diffrac. Suite Eva, Version 5.1.0.5*; Bruker AXS: Karlsruhe, Germany, 2019.
3. Moberly, J.G.; Bernards, M.T.; Waynant, K.V. Key features and updates for Origin 2018. J Cheminform **2018**, *10*, 5. https://doi.org/ 10.1186/s13321-018-0259-x
4. Bruker AXS. *TOPAS Version 5*, Technical reference. Bruker AXS, Germany, 2014
5. Lipson, H.; Langford, J.I.; Hu, H.-C. Trigonometric intensity factors. In *International Tables for Crystallography, Vol. C, Mathematical, physical and chemical tables*; Prince, E. (ed.), 3d ed.; International Union of Crystallography, Kluwer Academic Publ.: Dordrecht, Holland/Boston, USA, 2004, pp. 596–598.
6. Maslen, E.N. X-ray absorption. In *International Tables for Crystallography*, *Vol. C, Mathematical, physical and chemical tables*; Prince, E. (ed.), 3d ed.; International Union of Crystallography, Kluwer Academic Publ.: Dordrecht, Holland/Boston, USA, 2004, pp. 599–608.
7. Brown, A. G.; Fox, A.G.; Maslen, E.N.; O'Keefe, M.A.; Willis, B.T.M. Intensity of diffracted intensities. In *International Tables for Crystallography, Vol. C, Mathematical, physical and chemical tables*; Prince, E. (ed.), 3d ed.; International Union of Crystallography, Kluwer Academic Publ.: Dordrecht, Holland/Boston, USA, 2004, pp. 554–595.
